# Modelling heterogeneity in host susceptibility to tuberculosis and its effect on public health interventions

**DOI:** 10.1371/journal.pone.0206603

**Published:** 2018-11-14

**Authors:** Isaac Mwangi Wangari, James Trauer, Lewi Stone

**Affiliations:** 1 Mathematical Sciences, School of Science, Royal Melbourne Institute of Technology, Melbourne, Victoria, Australia; 2 School of Public Health and Preventive Medicine, Monash University, Melbourne 3004, Australia; 3 Biomathematics Unit, Department of Zoology, Faculty of Life Sciences, Tel Aviv University, Israel; Indian Institute of Technology Delhi, INDIA

## Abstract

A tuberculosis (TB) model that accounts for heterogeneity in host susceptibility to tuberculosis is proposed, with the aim of investigating the implications this may have for the effectiveness of public health interventions. The model examines the possibility that recovered individuals treated from active TB and individuals treated with preventive therapy acquire different levels of immunity. This contrasts with recent studies that assume the two cohorts acquire the same level of immunity, and therefore both groups are reinfected at the same rate. The analysis presented here examines the impact of this assumption when designing intervention strategies. Comparison of reinfection rates between cohorts treated with preventive therapy and recovered individuals who were previously treated for active TB provides important epidemiological insights. It is found that the reinfection rate of the cohort treated with preventive therapy is the one that plays the key role in qualitative changes in TB dynamics. By contrast, the reinfection rate of recovered individuals (previously treated from active TB) plays a minor role. Moreover, the study shows that preventive treatment of individuals during early latency is always beneficial regardless of the level of susceptibility to reinfection. Further, if patients have greater immunity following treatment for late latent infection, then treatment is again beneficial. However, if susceptibility increases following treatment for late latent infection, the effect of treatment depends on the epidemiological setting. That is: (i) in (very) low burden settings, the effect on reactivation predominates and the burden declines with treatment; (ii) in moderate to high burden settings the effect of reinfection predominates and burden increases with treatment. The effect is most dominant between the two reinfection thresholds, RT2 and RT1, respectively associated with individuals being treated with preventive therapy and individuals with untreated late latent TB infection.

## Introduction

Tuberculosis (TB) is a bacterial disease whose primary aetiological agent, *Mycobacterium tuberculosis* (*Mtb*) has become one of the most critical challenges to modern public health. *Mtb* is an ancient pathogen that has plagued humanity since antiquity [1–4] and has eluded all attempts to control it. Despite this long history and the fact that effective therapies for TB have been available for decades, it is currently estimated that a quarter of the world’s population is infected with TB [5] and ten to eleven million new cases of active disease emerge every year [6]. As of 2016, data from the World Health Organization show that TB is now the leading cause of death worldwide [6, 7], responsible for a staggering 1.7 million deaths per year globally.

After coming into contact with *Mtb*, individuals may progress directly to active infectious disease (primary progression) or enter a state of latent *Mtb* infection (LTBI) from which they may develop active disease after a variable period of time through “reactivation”. This pattern is consistent with epidemiological evidence indicating that the risk of active TB is highest in the first five years from exposure and declines thereafter, with the highest risk period being immediately after infection [8]. The risk of reinfection or superinfection with further episodes of exposure to *Mtb* is unclear, and although there is likely to be some degree of immunity to subsequent infections, little is known about the extent of protection [9, 10]. Models emphasize that understanding the degree of reduction of TB risk following previous infection in comparison to primary infection is critical to understanding the epidemiology of TB [8]. For example, following introduction of a drug-resistant *Mtb* strain into a population where TB burden is high, the proliferation of the strain may be hampered by the size of the effective susceptible population, which may be largely determined by the level of immunity among individuals with LTBI [11]. As a consequence, if latent infection provides sufficient protection against future infection, then the rate of infection with the resistant strain will fall, markedly curtailing the TB epidemic. However, issues such as disparities in infection rates between communities burdened with human immuno-deficiency virus (HIV) make it difficult to study reinfection directly, as the detrimental impact of HIV on immunity surpasses immunity provided by latent infection [8]. There have been past attempts to estimate the risk of reinfection amongst latently infected individuals, including through population models such as [12–15], which have shown risk reductions ranging from 41-81%. Together, these studies suggest that partial but substantial protection (from TB) is provided against future episodes of disease.

Besides reinfection of LTBI, individuals who have had active TB but have recovered, are also at risk of reinfection. For this reason, many models incorporate a compartment accounting for recovered individuals who remain susceptible to further episodes of TB (recurrent TB). It is important to note that there are two mechanisms by which recurrent TB can occur: (i) relapse with the previously responsible strain or (ii) reinfection from a new strain of TB. The latter contribution of exogenous reinfection with *Mtb* (in comparison to the endogenous reactivation of LTBI) to recurrent TB is a subject that is still debated as the two mechanisms cannot be easily disentangled [16]. However, advances in clinical medicine and gene technology, such as DNA fingerprinting techniques, can now distinguish the first episode of TB from the second [17–20]. Further, these techniques can determine whether a new episode of TB is caused by infection with the same strain as previously or a newly encountered strain, enabling classification of TB episodes as either relapse or reinfection, respectively. However, there is no consensus on whether recovered/treated individuals should be assigned a higher, lower or equivalent rate of infection in comparison to either latently infected or to uninfected individuals (susceptible). This raises the important question of how different levels of susceptibility across a population may interact to affect *Mtb* transmission dynamics. Some different approaches to exploring the impact of rates of recurrent TB adopted in the past include: assuming recovered individuals have no risk of reinfection [21, 22]; assuming relapse is responsible for all recurrent cases [23]; assuming equal risk of reinfection as for latently infected individuals [24]; assuming recovered individuals have equal rates of reinfection as for susceptible individuals [25]; incorporating both reinfection and relapse pathways after treatment [26]. Therefore, there is no consensus on whether recovered individuals have no risk of future infection, reduced risk, equal risk, or increased risk.

A previous review of recurrent TB episodes revealed that the proportion of recurrent cases that were due to subsequent infection with a new strain as opposed to relapse with the same strain varied markedly from 0-100% [27]. The review emphasized that relapse and reinfection should be treated as separate mechanisms and the two mechanims are likely to be responsible for the extent of variability in results. According to [28–31], rates of reinfection after successful treatment have been found to be variable in highly endemic regions, which likely reflects the degree of continuing exposure after treatment. Estimates of rates of recurrent TB in various settings often reach several thousand per 100,000 person-years, including estimates as high as 7850 per 100,000 persons-years [32]. A meta-analysis of such studies found that reinfection rates after successful treatment are higher than the background rate of TB in the community [33].

Currently, drugs are available that can be used to treat both individuals with LTBI and individuals with active TB, with the two most important first-line drugs being isoniazid and rifampcin. These two medications are effective in the treatment of active TB disease and as preventive therapy for patients who have previously been infected but are yet to manifest symptoms. Isoniazid preventive therapy (IPT) is the most commonly used preventive regimen globally and has established efficacy in dramatically reducing a patient’s future risk of progression to active TB [34]. Past case studies of isoniazid preventive therapy (IPT) among latently TB infected individuals (conducted in South Africa gold mines) suggested that IPT is effective at the individual level, significantly reducing the risk of subsequent diseases. However, the effect of IPT may be lost immediately when treatment is discontinued, which led the authors to conclude that the role of IPT at the population level is unclear. However, they also called for further research, since the effectiveness at the population level may have been compromised by a number of factors, such as post-treatment reinfection of miners or inadequately treated LTBI [35]. Other factors such as a high prevalence of HIV and silicosis, which are known to be strong risk factors for tuberculosis, may have also influenced the population level effect of IPT. Therefore, for IPT to be effective, it may need to be administered continuously amongst individuals at highest risk of TB.

Although, previous studies have considered population-level heterogeneity in susceptibility to reinfection between previously treated and latently infected persons [16, 36, 37], no previous work has considered differential susceptibility across all four possible exposure and treatment histories (i.e., fully susceptible, LTBI, treated LTBI and treated TB disease), together with the population level impact of all relevant public health interventions. Moreover, it is highly likely that the levels of susceptibility of the two previously treated populations differ considerably, given the likelihood that those treated for latent infection may retain some of the considerable immunological protection conferred by this infection, whereas the level of protection conferred by previous active TB is highly uncertain. In this study a TB model is presented with the aim of investigating: (i) how variability in risk of reinfection alters TB dynamics in a model accounting for heterogeneity in host susceptibility; (ii) how this variability in risk of reinfection influences the effectiveness of public health interventions.

## Model description

Following contact with *Mtb* an individual may develop TB disease as a result of one of three possible routes. These are fast primary progression after a recent infection, endogenous reactivation of LTBI and exogenous reinfection of a previously infected individual [38]. Here a deterministic mathematical model of the transmission of *Mtb*, taking into consideration the treatment of latently infected individuals with IPT is developed. Numerous infectious diseases demonstrate considerable latent periods during which an individual harbours the disease but does not manifest symptoms and is not infectious. A key feature of TB is its long latency period. This characteristic has crucial epidemiological implications [23], and thus most mathematical models of *Mtb* transmission in the literature incorporate latent compartments [39]. Through clinical observation it has been noted that following infection with TB, different rates of progression to active TB exist and that these rates decrease with time from infection. For example, 12.9% of patients with infection confirmed with interferon-gamma release assays following exposure to a smear-positive index case progressed to active TB in 23 months [40]. By contrast, after the initial high risk period, the rate at which reactivation TB occurs is relatively low and is estimated at 5-10% over 20 years [23]. To account for these marked differences, past mathematical models devoted to tracking TB dynamics have incorporated two major pathways from susceptible to actively infected: fast and slow TB progression. In such models, a fraction of exposed susceptibles progresses directly to active TB, bypassing the latency compartment [11, 23, 41, 42]. This modelling method enables a slight modification of the standard exponential function that governs time spent in the exposed compartment [43]. Other approaches include employing a stepwise reduction in the rate of progression occurring five years after exposure [14] or an arbitrary distribution of the latent period [41, 44].

In recent TB transmission models, compartments for both early and late latency are increasingly utilized to account for high and low risk periods following infection [16, 36, 45–47]. In such compartmental configurations all individuals progress to the early latent compartment following infection, after which a fraction may progress to infectious TB while the remainder transit to the low-risk late latent compartment [22, 47–49]. In consideration of the above discussion, the present study stratifies latent *Mtb* infection into two cohorts: a cohort at high risk of developing active TB, which is referred to as early LTBI, and a later stage of individuals with low risk for developing active TB, which shall be referred to as late LTBI. Therefore, the overall population is partitioned into six mutually exclusive classes: susceptible S which comprises individuals who have not come into contact with tuberculosis; early latently infected L_1_ which represents individuals who have recently been infected with *Mtb* (generally within a period of less than two years); late latently infected L_2_ which represent individuals with persistent latent TB who have contained TB infection and whose TB infection remains inactive; infectives I which represents individuals with active TB and are capable of infecting others; P which represents individuals who are being or who have been treated with isoniazid preventive therapy; recovered R which represents individuals who were previously infected and have been successfully treated. The total population is assumed to be large enough to be modelled deterministically and random mixing is assumed.

For the sake of mathematical tractability, here it is assumed that the birth rate compensates for TB-induced and background mortality (similar to the simplification used in some of the classical studies in the field, as for example in some of the key studies of Blower et al. [21, 23], Ziv et al. [47] and Dye et al. [50]). Thus, λ = *μ* + *dI* is the recruitment rate and all state variables are expressed as a fraction of the total population. The susceptible population comprises individuals who enter into this compartment at a rate λ and they diminish as individuals are infected with *Mtb* at a density-dependent infection rate *βI*, where *β* is the transmission coefficient. Newly infected individuals enter the early latent compartment L_1_ and it is assumed that a proportion of individuals in the early latent compartment are detected following screening for TB and are treated with IPT at rate *θ*, progressing to compartment P. A proportion of individuals in the early latent compartment progress to the active TB compartment I at a rate *fϕ*, while the remaining proportion proceeds to the late latent compartment at a rate (1 − *f*)*ϕ*.

Individuals in the late latent compartment may also receive IPT and thus progress to compartment P at a rate *ρ*. Furthermore, individuals in the late latent compartment can transit into the infectious compartment I due to endogenous reactivation of their latent TB at a rate *η*.

Only persons in the I compartment are infectious, and as such compartments L_1_, L_2_ and P do not contribute to the force of infection. Therefore, the infectious compartment is generated by fast progression of TB, endogenous reactivation from late latency and relapse of recovered individuals at a rate *ω*. The subpopulation is diminished when individuals are successfully treated at rate *τ* or as a result of spontaneous recovery (self cure) at a rate *α*.

Previously infected individuals may be fully susceptible to exogenous reinfection and infected at the same rate as the susceptible population (*S*(*t*)), or partially immune or have no immunity against reinfection. Consequently, late latently infected individuals, individuals treated with IPT and recovered individuals are reinfected at rates *σ*_*i*_ *β* (where i = 1, 2, 3), respectively, with *σ*_*i*_ ∈ [0, 1](*i* = 1, 2, 3) accounting for partial immunity against exogenous reinfection. Note that *σ*_*i*_ = 1(*i* = 1, 2, 3) corresponds to a scenario where late LTBI, treated LTBI and recovered individuals are infected at the same rate as susceptible individuals, while *σ*_*i*_ > 1(*i* = 1, 2, 3) implies that all post-infection cohorts have increased susceptibility to reinfection in comparison to susceptible individuals. This would also correspond to some past studies which have shown that individuals who have recovered from TB infection are more susceptible to future infection and in such a scenario *σ*_*i*_ > 1, (*i* = 1, 2, 3) [33].

**Fig 1:**
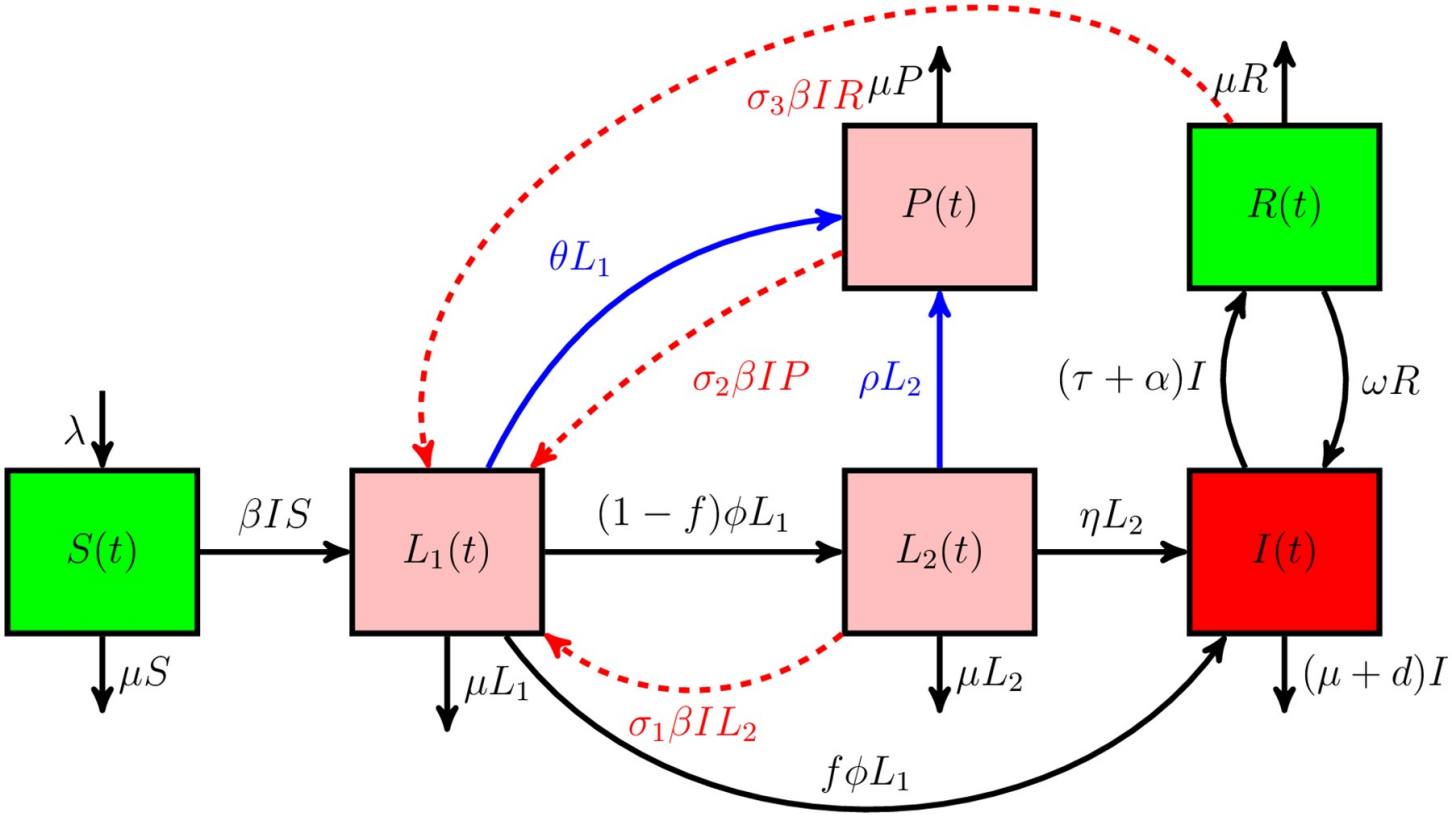
Schematic representation of the model. The square boxes represent classification of the general population into six mutually exclusive subpopulations, i.e., susceptibles *S*(*t*), early latents *L*_1_(*t*), late latents *L*_2_(*t*), individuals treated with isoniazid preventive therapy *P*(*t*), individuals with active TB *I*(*t*), and recovered individuals *R*(*t*). All arrows indicate either inflow or outflow or transition between compartments. Blue arrows illustrate transition of latently infected individuals as a result of treatment with IPT. Red dashed arrows show reinfection of late latently infected individuals, individuals treated with IPT and recovered individuals, respectively represented by *σ*_*i*_, *i* = 1, 2, 3.

All individuals experience natural death at a constant rate *μ*, including infectious individuals who suffer an additional TB-induced death at rate *d*. Transitions between compartments are shown diagrammatically in Fig 1. Combining the aforementioned assumptions, the following system of nonlinear ordinary differential equations govern the model:

$$\begin{array}{cc} \frac{dS}{dt} & =\lambda -\mu S-\beta IS,\\ \frac{dL_{1}}{dt} & =\sigma_{1}\beta IL_{2}+\sigma_{2}\beta IP+\sigma_{3}\beta IR+\beta IS-\left(\theta +\mu +\phi \right)L_{1},\\ \frac{dL_{2}}{dt} & =\left(1-f\right)\phi L_{1}-\left(\mu +\eta +\rho +\sigma_{1}\beta I\right)L_{2},\\ \frac{dI}{dt} & =\phi fL_{1}+\eta L_{2}+\omega R-\left(\mu +d+\tau +\alpha \right)I,\\ \frac{dP}{dt} & =\theta L_{1}+\rho L_{2}-\left(\mu +\sigma_{2}\beta I\right)P,\\ \frac{dR}{dt} & =\left(\tau +\alpha \right)I-\left(\mu +\omega +\sigma_{3}\beta I\right)R. \end{array}$$ (1)

The proposed model equations are different from the recently published model by Ragonnet et al. [46], in that each reinfection pathway is explicitly distinguished and individuals treated with IPT are not distinguished according to their time since infection. Moreover, since both early and late latently infected individuals are treated with isoniazid preventive therapy, instead of having two compartments for each as in [46], in this study the two compartments are coalesced into a single compartment for parsimony. Another important paper [36] incorporated treatment of early and late latent individuals but assumed that individuals treated with IPT and recovered individuals have identical risks of reinfection after recovery (i.e., *σ*_2_ = *σ*_3_). In the present study this assumption is relaxed by adding another compartment of individuals treated with IPT, so that the risk of exogenous reinfection can be varied between late latently infected individuals, individuals treated with IPT and recovered individuals. The motivation behind this is that there is a reasonable estimate of the value of *σ*_1_ (see [8, 16, 36, 51–53]), whereas *σ*_2_ and *σ*_3_ are highly uncertain.

The parameter values used in investigating the aforementioned objectives are selected from the relevant literature on TB epidemic models. The natural death rate *μ* is set to correspond to an average lifespan of 70 years [36]. From [54], the duration of TB from the first onset of TB symptoms to treatment or death is approximately three years. Consequently, both parameter *d* and *α* are estimated by assuming that *d* + *α* = 1/3 and 2*d* ≈ *α*. Thus, *d* is taken as *d* = 1/9 ≈ 0.1 From evidence that about 5-10% of the infected population manifest active TB shortly after infection [55, 56], parameter *f* is set to 0.05-0.1. Parameter *ϕ* is selected from a range of values *ϕ* ∈ [1.5, 12] [16, 36, 46, 47, 56]. The rate of endogenous reactivation among untreated late latent individuals is taken as *η* = 0.0002 per year, relapse among those who were previously cured through either therapeutic interventions or spontaneous cure is set to *ω* = 0.00002 per year; both adopted from [12, 14]. The relative risk of reinfection among untreated late latent individuals, *σ*_1_ is fixed at 0.25 as in [36, 51], with the justification that it agrees with the maximum level of immunity rendered by BCG (Bacille Calmette-Guérin) vaccination [57] (although the effects of varying this parameter from its baseline value are explored in detail below). The parameter *σ*_2_ corresponds to the relative risk of reinfection among individuals treated with IPT, while *σ*_3_ corresponds to the relative risk of reinfection among recovered individuals. Exploring the effects of varying these highly uncertain parameters (including their epidemiological effects and their influence on the effectiveness of public health interventions) is the primary purpose of this study. The baseline parameter value for therapeutic intervention among individuals manifesting TB symptoms is set at *τ* = 2 per year, which corresponds to a mean duration of infectiousness of six months [36] (which implicitly assumes that the R compartment incorporates those currently under treatment for active disease). Last, the transmission coefficient *β* is varied over a wide range. A summary of the parameters and their respective values are shown in Table 1.

**Table 1:** Parameters and definitions for model Eq (1).

Parameter	Definition	baselinevalue	range	References
*β*	Transmission coefficient	–	0-500 *yr*^−1^	[16, 36]
*μ*	Natural death rate	1/70 *yr*^−1^	–	[16, 36, 46, 47]
*d*	TB-induced death rate	0.1 *yr*^−1^	- -	[41, 54]
*ϕ*	Rate at which infected individuals exit early latent compartment *L*_1_	12 *yr*^−1^	1.5-12	[16, 36, 46, 47]
*f*	Fraction of TB infected population that progress to active TB soon after infection	0.05	0.05-0.1	[55]
*η*	Rate of endogenous reactivation for late latents	0.0002 *yr*^−1^	- -	[24, 58]
*τ*	Treatment rate of active TB	2 *yr*	- -	[16, 36, 59]
*α*	Spontaneous cure/self cure	2/9 *yr*^−1^	- -	-
*θ*	Treatment rate of LTBI *L*_1_ with IPT	1	variable	[36]
*ρ*	Treatment rate of LTBI *L*_2_ with IPT	0.1	variable	[36]
*ω*	Rate of relapse following recovery	0.00002 *yr*^−1^	- -	[12, 14]
	**Levels of susceptibility**			
*σ*_1_	Multiplier for exogenous reinfection for latent *L*_1_	0.25	0.25-1	[8, 16]
*σ*_2_	Multiplier for exogenous reinfection for population treated with IPT	0.5	0.25-2	[8, 16, 33]
*σ*_3_	Multiplier for exogenous reinfection for recovered population	0.5	0.25-2	[8, 16, 33]

## Basic reproduction number *R*_0_

In epidemic theory the basic reproduction number, denoted by *R*_0_, is one of the most important model quantities, given its ability to predict the triggering of an epidemic. *R*_0_ is defined as the number of secondary infections that would occur when a single infectious individual is introduced into an entirely susceptible population, and considered over the lifetime of the disease. Following the method in [60] the basic reproduction number for the model system (1) as computed in S1 Appendix is given as:

$$\begin{array}{c} R_{0}=\frac{\beta (\mu +\omega )\phi (f(\mu +\rho )+\eta )}{\left((\mu +d)(\mu +\omega )+\mu (\tau +\alpha ))(\mu +\eta +\rho )(\theta +\mu +\phi \right)}. \end{array}$$ (2)

(See S2 Appendix for biological interpretation of this expression (2) for *R*_0_.) In general it is known that a value of *R*_0_ < 1 implies that each individual is only able to infect less than one individual on average, such that the disease will die out, whereas a value of *R*_0_ > 1 implies that each individual is able to infect more than one individual and that endemic disease will persist within the population. Hence, *R*_0_ = 1 is a crucial epidemic threshold in determining the epidemic trajectory.

**Fig 2:**
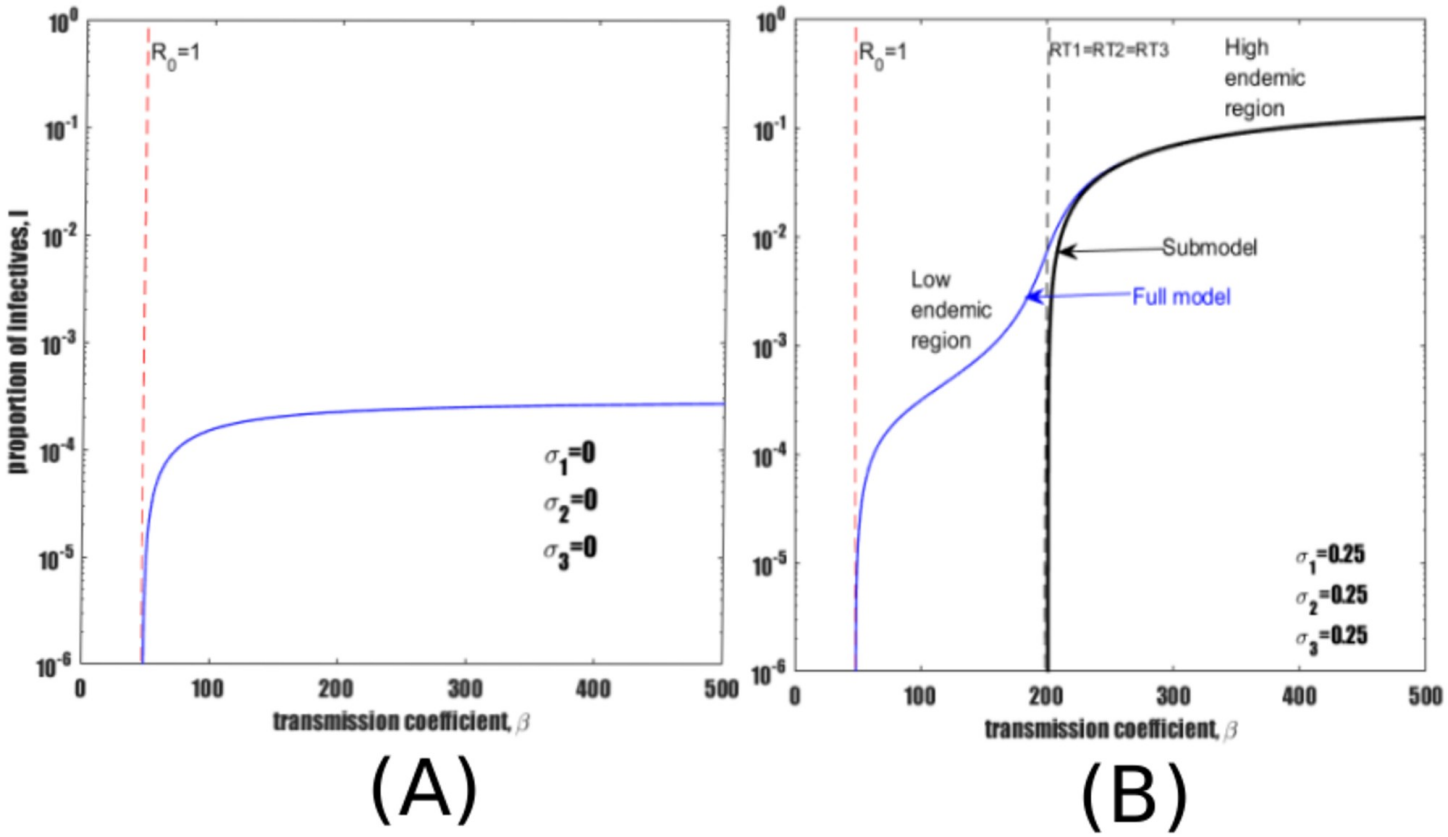
Bifurcation diagram of equilibrium TB prevalence I* as a function of *β*. (A) In the absence of reinfection the epidemic threshold occurs at *R*_0_ = 1. (B) Bifurcation diagrams of the full model equilibria (See S3 Appendix) and reinfection submodel (3) equilibria superimposed on the same axes. In both figures the red dotted vertical line marks the bifurcation point *R*_0_ = 1. Black vertical dashed line marks the point where reinfection thresholds RT1, RT2 and RT3 converge. Parameters as in Table 1. In both panels semi-logarithmic scale is used for clarity.

Fig 2A illustrates that in the complete absence of reinfection pathways (*σ*_1_ = *σ*_2_ = *σ*_3_ = 0), the model dynamics are quite simple. When *R*_0_ < 1 there is a disease free equilibrium (I* = 0), or DFE, and when *R*_0_ > 1 there is one endemic equilibrium. (Note the logarithmic scale in the figure which hides the DFE).

## Reinfection thresholds

With the introduction of reinfection pathways, the non-linear dynamics of model Eq (1) yield a different bifurcation structure. Besides the threshold at *R*_0_ = 1 (red dotted line), there are other “reinfection thresholds” which play a critical role in determining endemic equilibria [36, 51]. They are typified by the blue endemic curve in Fig 2B, which is obtained by plotting I* versus the transmission coefficient *β* for the full model (1). The figure illustrates the existence of a low endemic region (low I* for *β* < 200) and a high endemic region (high I* for *β* > 200). As is typical, disease prevalence increases by two orders of magnitude when transmission increases across the reinfection threshold [51, 52]. [51, 52] introduced a technique for determining reinfection thresholds, The thresholds were shown to occur when rates of reinfection are just sufficient to maintain an endemic disease in the absence of contributions from other pathways (i.e., primary infection and reactivation mechanisms).

Using this technique, the reinfection thresholds of model (1) can be approximated by analysing special submodels that distinguish reinfection from other transmission processes such as primary infection, reactivation and relapse [61]. The first step requires setting endogenous reactivation and relapse to zero (i.e., *βIS* = 0 and *η* = *ω* = 0). It is then possible to calculate three reinfection thresholds from the following respective submodels:

1. RT1: The threshold due to reinfection during late latency (*L*_2_). In this scenario, reinfection of recovered individuals and those previously treated for latent infection are switched off (i.e., *R*(*t*) = *P*(*t*) = 0);
2. RT2: The threshold due to reinfection of individuals previously treated for LTBI (*P*). In this scenario, reinfection of recovered individuals and those with latent infection are switched off (*R*(*t*) = *L*_2_(*t*) = 0);
3. RT3 The threshold due to reinfection of recovered individuals (R). In this scenario, reinfection of latently infected individuals and those previously treated for LTBI are switched off (i.e., *L*_2_(*t*) = *P*(*t*) = 0).

The following example gives calculations for finding the first reinfection threshold RT1 in model (1). As mentioned, reactivation and primary infection mechanisms are set to zero (*βIS* = 0 and *η* = *ω* = 0), and post-infection levels of population immunity risk are assumed to be homogeneous or equal (that is *σ*_1_ = *σ*_2_ = *σ*_3_). In this configuration, the rates of infection of compartments L_2_, P and R become equivalent, so that it is possible to merge these three compartments. Using the procedure outlined in [61], the reinfection submodel is

$$\begin{array}{cc} \frac{d(L_{2}+P+R)}{dt} & =\lambda +\left(1-f\right)\phi L_{1}+\left(\tau +\alpha \right)I+\theta L_{1}-\mu \left(L_{2}+P+R\right)-\sigma_{1}\beta I\left(L_{2}+P+R\right),\\ \frac{dL_{1}}{dt} & =\sigma_{1}\beta I\left(L_{2}+P+R\right)-\left(\theta +\mu +\phi \right)L_{1},\\ \frac{dI}{dt} & =\phi fL_{1}-\left(\mu +d+\tau +\alpha \right)I. \end{array}$$ (3)

The Jacobian matrix of the reinfection submodel (3) evaluated at the disease free equilibrium (1, 0, 0) is then

$$\begin{array}{c} J_{R}=(\begin{array}{ccc} -\mu & (1-f)\phi +\theta & \left(d+\tau +\alpha \right)-\sigma_{1}\beta \\ 0 & -(\theta +\mu +\phi ) & \sigma_{1}\beta \\ 0 & \phi f & -(\mu +d+\tau +\alpha ) \end{array}). \end{array}$$ (4)

Setting the determinant of the Jacobian matrix (4) to zero yields the critical value of the first reinfection threshold,

$$\begin{array}{c} \beta =\frac{1}{\sigma_{1}}\frac{\left(\theta +\mu +\phi )(\mu +d+\tau +\alpha \right)}{\phi f}=\text{RT}1. \end{array}$$ (5)

We set parameters *ϕ* = 12 and *θ* = 1 while others remain as shown in Table 1. This yields RT1 ≈ 200 (see Fig 2B where RT1 is marked with a black dotted line). The reinfection threshold expressed in terms of *R*_0_ is obtained by substituting (5) into Eq (2), leading to

$$\begin{array}{c} R_{0}^{\text{RT}1}=\frac{1}{\sigma_{1}}\frac{\left(\mu +d+\tau +\alpha )(\mu +\omega )(f\phi (\mu +\rho )+\eta \phi \right)}{f\phi ((\mu +d)(\mu +\omega )+\mu (\tau +\alpha ))(\mu +\eta +\rho )}. \end{array}$$ (6)

Note that, if the reactivation and relapse mechanisms are now set equal to zero (*ω* = *η* = 0) then expression (6) reduces to *R*_0_ ≈ 1/*σ*_1_ which is equivalent to the simplest form of reinfection threshold in terms of *R*_0_, as originally obtained by Gomes et al. [51]. The other reinfection thresholds RT2 and RT3 are computed similarly as shown in S4 Appendix. The equilibrium of the reinfection submodel (3) can be easily obtained by setting the right-hand terms to zero and evaluating for I* as

$$\begin{array}{c} I^{*}=\frac{f\phi [\beta -\frac{\left(\theta +\mu +\phi )(\mu +d+\tau +\alpha \right)}{\sigma_{1}f\phi }]}{\beta [f\phi +(\mu +d+\tau +\alpha )]}. \end{array}$$ (7)

(See S4 Appendix for other steady states).

Fig 2B shows that the submodel approximates the behaviour of the full model in the vicinity of the reinfection threshold RT1. The black solid endemic curve in Fig 2B represents the equilibrium value of I* (see Eq (7)) for the submodel Eq (3) as a function of *β*. Above *β* = 200 there is a positive endemic equilibrium but below this value only the disease-free equilibrium is present. Thus, the theoretically predicted reinfection threshold for the submodel (3) is confirmed to be RT1 = 200. There is a transition from low to high TB burden with the proportion of active TB increasing by about two orders of magnitude when *β* increases beyond *β* ≈ 200 (as can also be observed in [36, 51]).

## The impact of reinfection parameters *σ*_*i*_

### Interpretation of risks of reinfection

First, it is useful to reflect on how the reinfection parameters *σ*_*i*_(*i* = 1, 2, 3) should be interpreted by re-examining a typical infection term *σ*_*i*_ *β* in Eq (1). By *σ*_*i*_ < 1; (*i* = 1, 2, 3) accounting for partial immunity against exogenous reinfection among post-infection cohorts. *σ*_*i*_ = 1(*i* = 1, 2, 3) corresponds to a scenario where late LTBI, treated LTBI and recovered individuals are infected at the same rate as susceptible individuals. *σ*_*i*_ > 1(*i* = 1, 2, 3) implies that all post-infection cohorts have increased susceptibility to reinfection in comparison to susceptible individuals. That is individuals who have already been infected have higher risk of reinfection, when compared with a typical susceptible individual who has never been infected. This indicates that individuals may have increased susceptibility to tuberculosis and is biologically plausible, e.g., due to local tissue damage to the respiratory tract impairing innate immunity. The latter would also correspond to some past studies which have shown that individuals who have recovered from TB infection are more susceptible to future infection [33].

### Homogeneous reinfection risk (*σ*_1_ = *σ*_2_ = *σ*_3_ < 1)

Fig 2B indicates a typical bifurcation diagram (marked by a blue endemic curve) for the case where *σ*_*i*_ < 1(*i* = 1, 2, 3). Here all cohorts susceptible to reinfection have equal risk of reinfection (*σ*_1_ = *σ*_2_ = *σ*_3_ = 0.25). This is our reference frame case. The reinfection thresholds RT1 = RT2 = RT3 ≈ 200 divide the transmissibility axis into low and high endemic regions. For *β* ⪅ 50 (equivalently *R*_0_ < 1), I* = 0. For 50 ⪅ *β* ⪅ 200 then I*≈10^−3^, when *β* ≈ 200 then I* ≈ 10^−2^ and when *β* > 200 then I* → 10^−1^.

### Heterogeneity in susceptibility to reinfection

**Fig 3:**
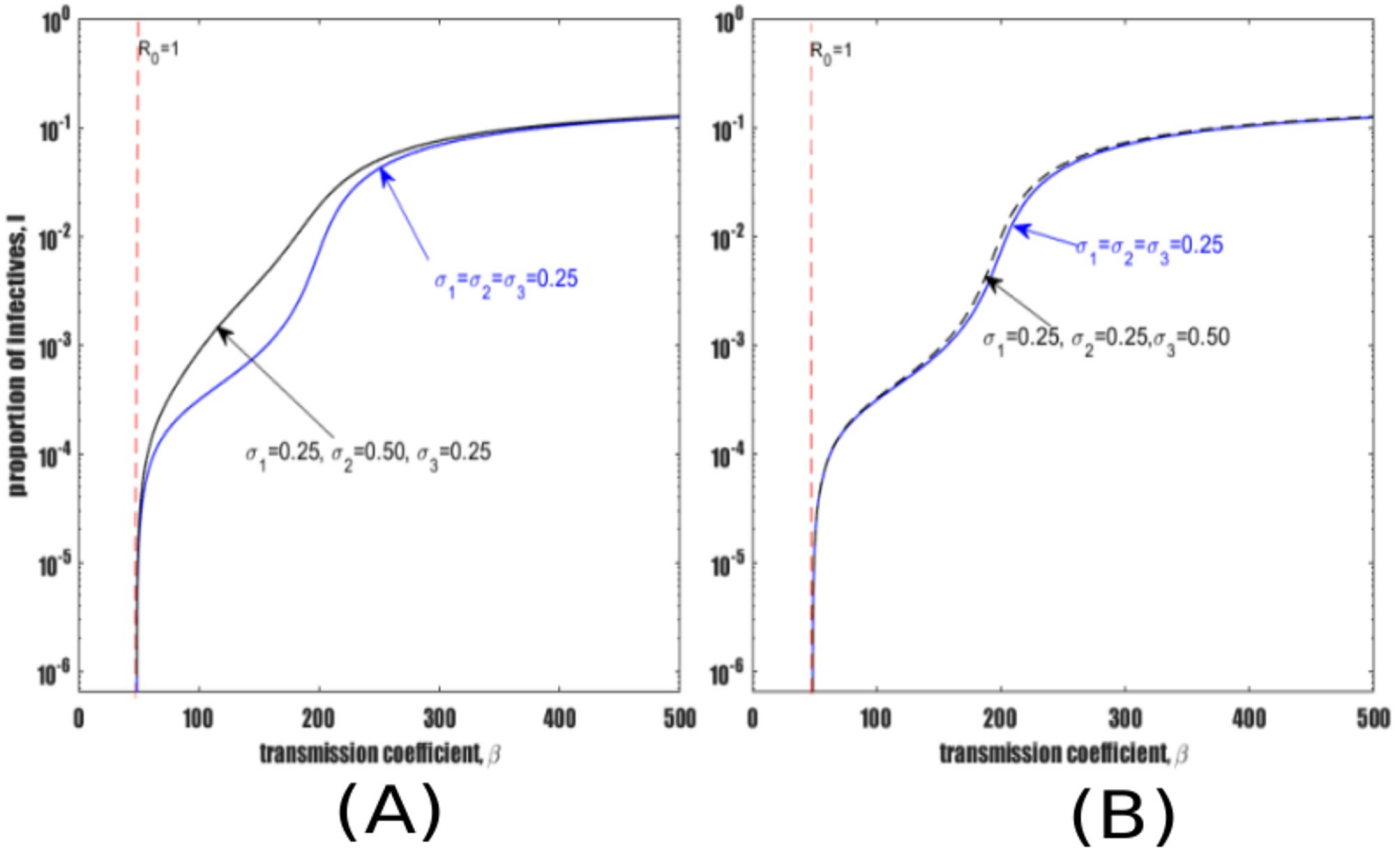
Illustration of the relative importance of *σ*_2_ and *σ*_3_ to equilibrium dynamics. Parameters as in Table 1. Reference homogeneous model *σ*_1_ = *σ*_2_ = *σ*_3_. (A) Impact of increasing *σ*_2_. (B) Impact of increasing *σ*_3_.

We now proceed to investigate how reinfection parameters *σ*_2_ and *σ*_3_ impact TB dynamics compared to the reference frame model for which *σ*_1_ = *σ*_2_ = *σ*_3_ = 0.25. Setting *σ*_1_ = 0.25, *σ*_2_ = 0.5, *σ*_3_ = 0.25 results in Fig 3A which shows that increasing *σ*_2_ results in an increase in TB prevalence compared to the reference model. On the other hand, setting *σ*_1_ = *σ*_2_ = 0.25 and *σ*_3_ = 0.5 results in Fig 3B which indicates that *σ*_3_ has little effect on TB prevalence in comparison to the same increase in *σ*_2_ (cf., Fig 3A).

**Fig 4:**
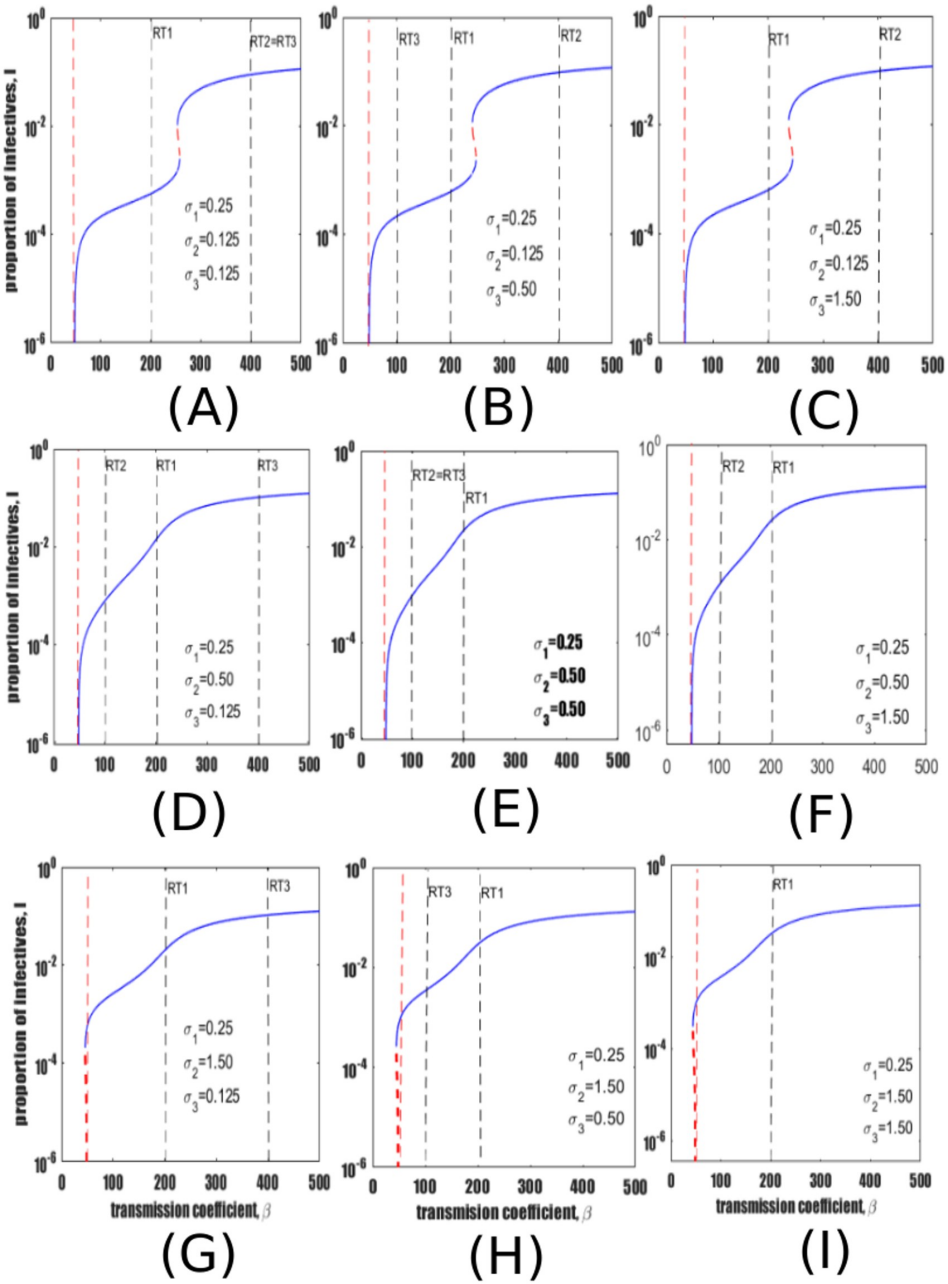
Exploration of the full range of plausible values of the relative risk of reinfection parameters. All panels show equilibrium prevalence as a function of the transmission coefficient *β*. From left to right, each column shows an increase in the relative risk of reinfection among recovered individuals *σ*_3_, while from top to bottom each row shows increasing risk of reinfection among individuals treated with IPT *σ*_2_. Other parameter values remain at baseline values in Table 1. *σ*_*i*_ values are presented such that individuals previously treated for LTBI or for active disease may have 1) lesser susceptibility than that estimated for LTBI (*σ*_2_, *σ*_3_ < *σ*_1_), 2) intermediate susceptibility between LTBI and full susceptibility (*σ*_2_ or *σ*_3_ > *σ*_1_, but < 1) or 3) greater susceptibility than full susceptible persons (*σ*_2_ or *σ*_3_ > 1). The red dotted vertical line marks the point at which *R*_0_ = 1. The dotted red segment of the endemic curves represents the unstable equilibria while the blue line represents stable equilibria.

Further model dynamics are explored over a wider range of reinfection parameters by again modifying the relative risks of reinfection among individuals treated with IPT (*σ*_2_) and those previously recovered from active TB (*σ*_3_). The relative rate of reinfection among LTBI is fixed to *σ*_1_ = 0.25, consistent with the pertinent literature described above and with [36, 51, 52]. The remaining two risk of reinfection parameters, (*σ*_2_ and *σ*_3_) are varied and may take values of 0.125, 0.50 and 1.5, thereby creating heterogeneity in susceptibility to TB transmission. From left to right the three columns of panels in Fig 4 show an increasing risk of reinfection among recovered individuals (*σ*_3_) while from top to bottom each row of the figure shows an increasing risk of reinfection among individuals treated with IPT (*σ*_2_).

It is observed that as *σ*_2_ increases (i.e., moving from top to bottom of each column in Fig 4), there is a structural change in the bifurcation curve, and TB prevalence rises. In contrast, within each row of panel 4 there is no significant qualitative change in the bifurcation structure as *σ*_3_ is varied (parameter values *σ*_3_ = 0.125, 0.50, 1.50) i.e., moving from left to right.

Moreover, considering a scenario where either individuals treated with IPT or recovered individuals (or both) have a significant loss of immunity by readjusting *σ*_2_ and *σ*_3_ such that they can take values greater than one results in bi-stability phenomena whereby TB can be endemic below the endemic threshold *R*_0_ = 1. This is observed in Fig 4. However, the occurrence of backward bifurcation is attributed to *σ*_2_ and not *σ*_3_ as illustrated in the last row of panels of Fig 4 where backward bifurcation sets in when *σ*_2_ > 1. By backward bifurcation we mean a situation where both stable and unstable equilibria coexist when *R*_0_ is less than one. More simulations show that *σ*_3_ has minimal effect as can be observed in Fig 4C and 4F appearing in the last column of panel Fig 4.

### Other features: Hysteresis

**Fig 5:**
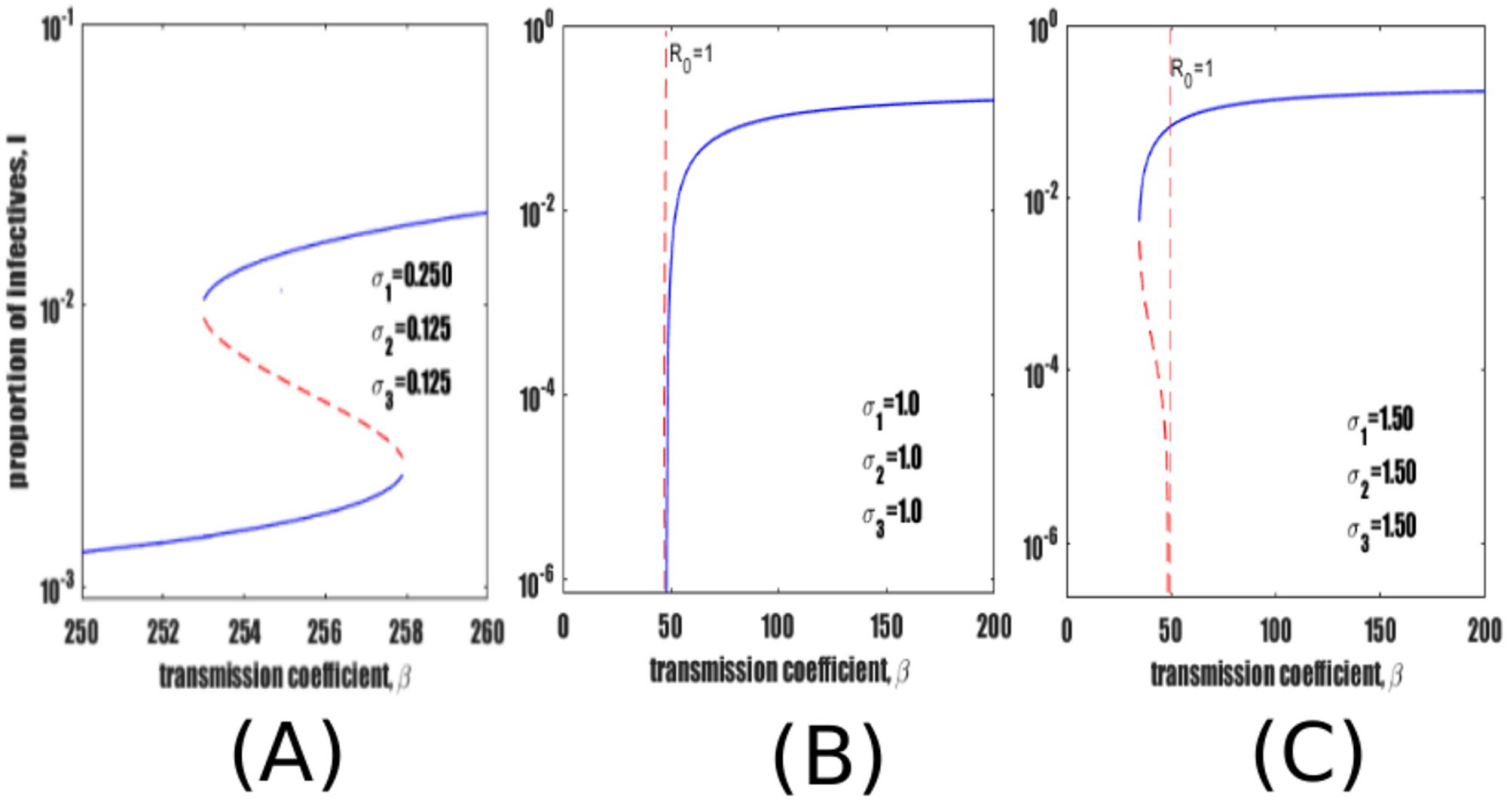
Emergence of hysteresis and backward bifurcation effects with changes to reinfection parameters. Parameters as in Table 1. (A) Occurrence of hysteresis. (B) No reinfection thresholds. All cohorts have immunity equal to susceptible population. (C) Backward bifurcation. All cohorts have no partial immunity and have increased susceptibility to reinfection compared to susceptibles.

Selecting parameters such that *σ*_2_ = *σ*_3_ = 0.125, and *σ*_1_ = 0.25 while other parameters remain at baseline values, results in Fig 5A, which shows a hysteresis phenomenon. Hysteresis implies that multiple equilibria, both stable and unstable, occur simultaneously above the epidemic threshold *R*_0_ = 1 (see [62, 63]). In Fig 5A the unstable equilibrium is marked by a red dotted line that separates two stable equilibria: low endemic and high endemic. Rather similar to a backward bifurcation, there can be jumps between the two stable equilibria. In the regime where the contact rate is approximately *β* = 253 there is a low endemic equilibrium. A small epidemiological change, such as a slight rise in *β*, (which pushes *I* above the unstable equilibrium marked by the red dashed line) may trigger a jump to the high endemic equilibrium.

### No partial immunity

As discussed, the degree of protection conferred by initial infection with TB is still controversial. Gomes et al. [16] and Verver et al. [33] suggest that the risk of reinfection parameters *σ*_1_, *σ*_2_, *σ*_3_ can in some cases be close to or greater than unity, so that individuals who have already been infected may have the same or even higher risks of reinfection as compared to typical susceptible individuals who have never been infected. As discussed above, this situation seems less intuitive, but remains plausible. For example, Gomes et al. [16] estimated *σ*_3_ = 0.51 with confidence interval of [0.00, 2.37] using a heterogeneous model, while with a homogeneous model *σ*_3_ = 3.87 with confidence interval of [1.61, 7.79].

Consider now a scenario in which all cohorts subject to reinfection have the same susceptibility to infection as susceptible individuals (note that this is equivalent to a classic SEIS model). That is, *σ*_1_ = *σ*_2_ = *σ*_3_ = 1. This leads to the bifurcation diagram in Fig 5B and demonstrates that TB will jump to a high endemic levels as soon as the basic reproduction number exceeds *R*_0_ = 1. Moreover, if all cohorts subject to reinfection have higher rates of infection than susceptible individuals, i.e., *σ*_*i*_ > 1, then the phenomenon of backward bifurcation occurs as shown in Fig 5C and TB can exist in a high endemic state on either side of the threshold *R*_0_ = 1.

## Impact of intervention on reinfection

### Effect of treating early LTBI

**Fig 6:**
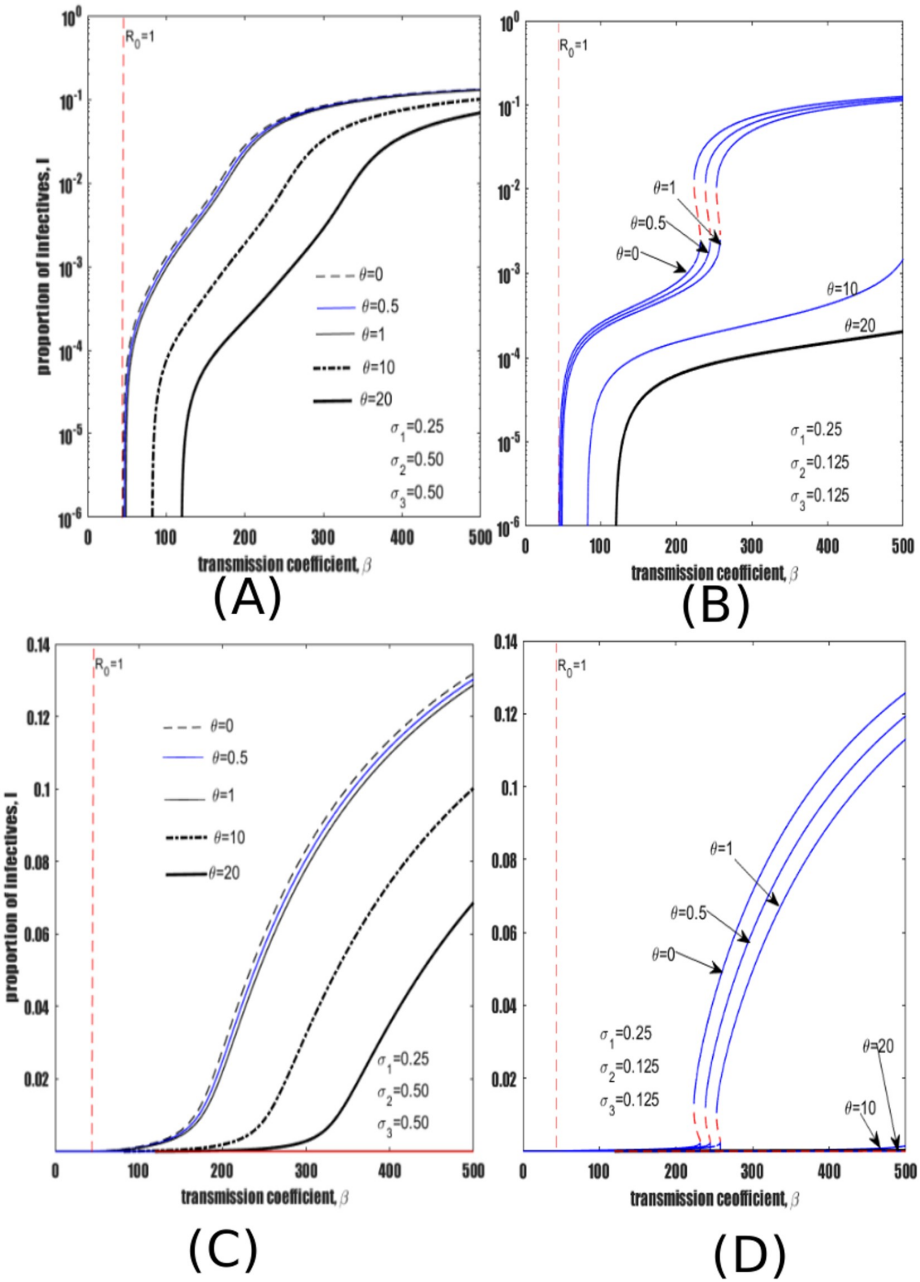
Impact of treating early latent individuals (*θ*) with IPT therapy under different risks of susceptibility to reinfection *σ*_1_. (A) Risks of reinfection among individuals treated with IPT and recovered individuals are greater than risk of reinfection of late LTBI. That is *σ*_2_, *σ*_3_ > *σ*_1_. (B) The level of susceptibility to reinfection among late LTBI is lower than for both individuals treated with IPT and individuals treated from active TB. (C) and (D) respectively represents figures (A) and (B) plotted in linear scale. Treatment of late LTBI and individuals with active TB are respectively set at *ρ* = 0.1 and *τ* = 2 while other parameters are as shown in Table 1.

The effect of treating early LTBI for different levels of susceptibility to reinfection is examined. Recall that treatment of early latent TB with preventive therapy (IPT) is modelled by parameter *θ*. First, assume that the relative rate of reinfection among late LTBI (*σ*_1_) is less than the levels of reinfection of both recovered individuals (*σ*_3_) and individuals treated with IPT (*σ*_2_). Consider the risk of reinfection parameters *σ*_2_ = *σ*_3_ = 0.5 and *σ*_1_ = 0.25. Fig 6A illustrates model dynamics for different values of the treatment parameter *θ*, and shows that treatment of early latent LTBI decreases TB prevalence regardless of the higher risk of reinfection (i.e., *σ*_2_ > *σ*_1_ and *σ*_3_ > *σ*_1_).

Now consider a scenario where the level of susceptibility to reinfection of late LTBI is high in comparison to the levels of susceptibility to reinfection of both recovered and individuals treated with IPT. Selecting *σ*_2_ = *σ*_3_ = 0.125 and *σ*_1_ = 0.25, yields the bifurcation diagram in Fig 6B. Again it is observed that treatment of early LTBI via *θ* decreases TB prevalence. These same results are plotted with linear scales in Fig 6C and 6D. Observe that the magnitude of TB reduction is relatively stronger when *θ* > 1. Note that values of *θ* of the order presented in Fig 6 may be plausible, given that the proportion of infections identified and treated immediately is given by *θ*/(*θ* + *ϕ* + *μ*) where (*ϕ* + *μ* ≈ 12).

### Effect of treating late LTBI

In a previous related study, Gomes et al. [36] investigated a scenario where intervention (i.e., treatment of late LTBI) is assumed to increase or decrease risks of reinfection i.e., the values of *σ*_*i*_. However this study did not distinguish between individuals treated with IPT and individuals previously assumed to have recovered due to antibiotic treatment or self-cure. Thus, contrary to Gomes et al. [36] where only two groups that were subject to reinfection were considered, the model presented here has three such cohorts and four levels of susceptibility to infection across the population. Distinguishing between individuals treated for active TB and individuals treated with IPT provides a more comprehensive analysis of treating late LTBI when the population is subjected to different levels of reinfection.

**Fig 7:**
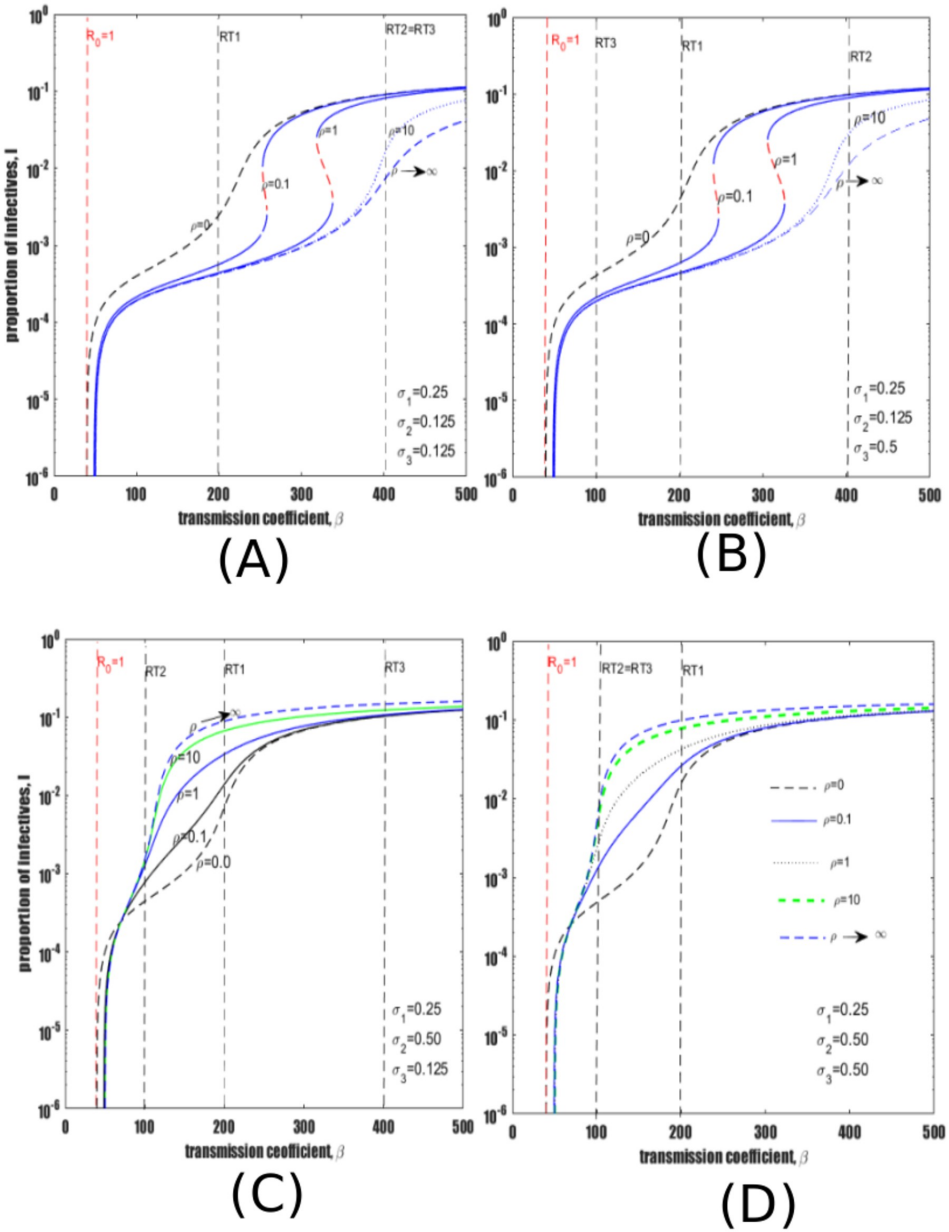
Effect of treatment for late latent infection under the assumption that treatment decreases (panels (A) and (B)) and increases (panels (C) and (D)) susceptibility to reinfection, and assuming that treatment for active disease decreases (panels (A) and (D)) and increases (panels (B) and (D)) susceptibility to reinfection. Treatment of individuals with active TB is fixed to *τ* = 2, and treatment of individuals with early latent TB is fixed to *θ* = 1, while treatment of late latent individuals is introduced at rates; *ρ* = 0, 0.1, 1, 10 and the limit *ρ* → ∞. Other parameters as shown in Table 1. (A) *σ*_2_, *σ*_3_ < *σ*_1_. (B) *σ*_2_ < *σ*_1_ < *σ*_3_. (C) *σ*_3_ < *σ*_1_ < *σ*_2_. (D) *σ*_1_ < *σ*_2_, *σ*_3_. The black dashed endemic lines represent the baseline case where there is no treatment of late LTBI (i.e., *ρ* = 0) while the blue dashed endemic lines represents immediate treatment for the entire population (*ρ* → ∞). In both figures (A) and (B) the dashed red lines of the endemic curves represent unstable equilibria while blue solid lines represent stable equilibria. All figures are plotted with semi-logarithmic scale.

Recall that parameter *ρ* represents treatment of late LTBI with IPT. Bifurcation diagrams obtained for different levels of reinfection of individuals treated with IPT (modified by *σ*_2_) and recovered individuals (modified by *σ*_3_) to reinfection of late LTBI (modified by *σ*_1_) are compared in Fig 7). Panels are again presented with *σ*_2_ increasing from top to bottom while *σ*_3_ increases from left to right. First, assuming intervention decreases the level of susceptibility to reinfection, then both recovered individuals and individuals treated with IPT may be given a lower rate of reinfection in comparison to late LTBI. As an example, we set *σ*_2_ = *σ*_3_ = 0.125 while *σ*_1_ = 0.25, and obtain the typical bifurcation diagram in Fig 7A. The baseline no-treatment scenario is obtained by setting *ρ* = 0 while the extreme case is illustrated by assuming *ρ* → ∞. Fig 7A illustrates that in a scenario where *σ*_2_, *σ*_3_ < *σ*_1_, treatment of late LTBI reduces TB prevalence. This decrease in TB prevalence is largely attributable to the general reduction in susceptibility to reinfection. In addition, Fig 7A indicates the existence of bistable equilibria (hysteresis effect) in which stable equilibria are separated by an unstable equilibrium (dashed red line).

Performing another simple numerical experiment can shed some light on the differences between reinfection parameters *σ*_2_ and *σ*_3_. Hence, assuming *σ*_2_ < *σ*_1_ and *σ*_3_ > *σ*_1_, and plotting TB prevalence as a function of *β* results in Fig 7B, which shows dynamics which are almost the same as Fig 7A. That is treatment of late LTBI remains beneficial despite *σ*_3_ = 0.5 being double *σ*_1_. These and similar numerical results imply again that *σ*_3_ is unimportant, while *σ*_2_ is the main parameter of interest. The respective reinfection thresholds associated with individuals treated with IPT (RT2), recovered individuals (RT3) and late LTBI (RT1) are marked on Fig 7 with black dotted lines. Considering the regions bounded by reinfection thresholds, it is clear from Fig 7B that treatment has the most beneficial impact within the region bounded by RT1 and RT2, and the position of RT3 has little influence.

Assume now that *σ*_1_ = 0.25, *σ*_2_ = 0.5 and *σ*_3_ = 0.125 so that the level of reinfection among individuals treated with IPT is relatively large. Such set of risk of reinfection parameters leads to Fig 7C which shows that for certain values of transmission coefficient *β*, treatment of late LTBI lead to an increase in TB prevalence. Again it is evident from Fig 7C that the reinfection thresholds RT2 and RT1 bound the parameter space where treatment of late LTBI has most impact. Outside this region treatment has a smaller effect.

Finally, assuming susceptibility to reinfection among individuals treated with IPT and recovered individuals increases after treatment, results in Fig 7D where *σ*_2_ = *σ*_3_ = 0.50 while *σ*_1_ = 0.25. Fig 7D again shows that increasing treatment of late LTBI may lead to an increase in TB prevalence when *σ*_2_, *σ*_3_ > *σ*_1_. Note that the greatest increase in TB prevalence also occurs between the reinfection thresholds RT2 (= RT3) and RT1.

All of these results imply that the relative magnitude of risk of reinfection *σ*_1_ and *σ*_2_ are vital in determining whether treatment will increase or decrease TB prevalence.

Gomes et al. [36] investigated the effect of treating late latent TB under two assumptions: a) susceptibility to reinfection increases after treatment of late LTBI, and b) susceptibility to reinfection decreases after treatment of late LTBI. They found that if treatment of late LTBI increases the risk of reinfection of recovered individuals *σ*_2_ then TB prevalence increases, while if treatment of LTBI is assumed to decrease risk of reinfection of recovered individuals *σ*_2_, then TB prevalence decreases [36]. As mentioned above, in Gomes et al. [36] both individuals treated for active TB and individuals treated with preventive therapy were assumed to be indistinguishable and therefore classified as one cohort of recovered individuals.

**Fig 8:**
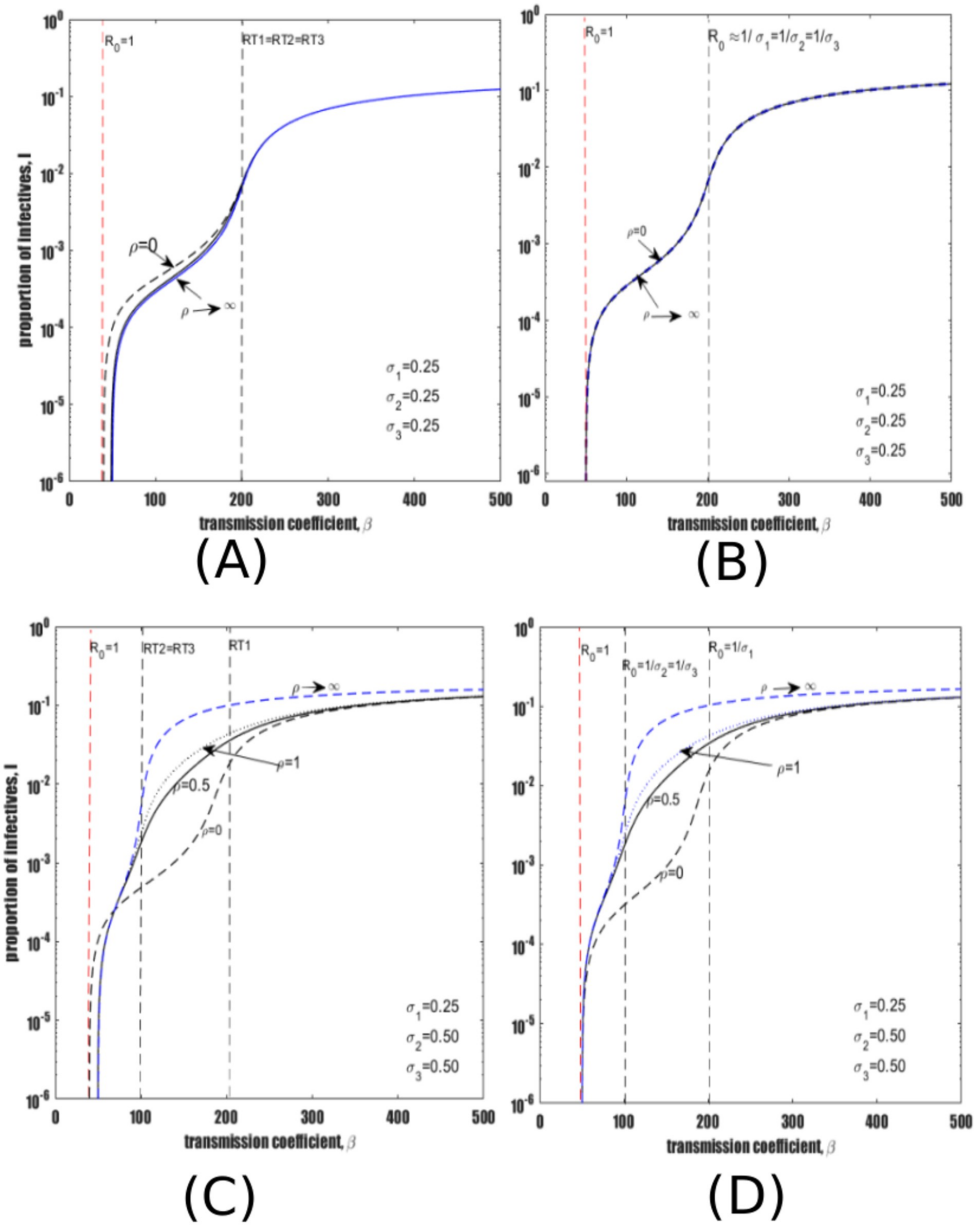
Treatment of late latent infection under the assumption of no effect on susceptibility to reinfection (panels (A) and (B)) and increased susceptibility (panels (C) and (D)), with reactivation mechanisms present (panels (A) and (B)) and removed (panels (B) and (D)). Parameters used are *τ* = 2, *θ* = 1, *ρ* = 0, 0.5, 1 and *ρ* → ∞ while other parameters remain as in Table 1. The risks of reinfection used are shown in each figure.

Further, it is important to investigate a scenario where latently infected individuals have the same level of reinfection as individuals treated with IPT and recovered individuals. Thus, considering *σ*_1_ = *σ*_2_ = *σ*_3_ = 0.25 while varying treatment results in Fig 8A which depicts that treatment has a positive impact between the region bounded by *R*_0_ = 1 and RT1 (= RT2 = RT3) and minimal impact above the reinfection thresholds RT1 (= RT2 = RT3). Fig 8B is obtained using the same parameter values as Fig 8A except that the reactivation mechanisms are switched off (i.e., *ω* = *η* = 0). It is observed that all the endemic curves under different treatment values merge. However, further exploration of a scenario where reactivation mechanisms are neglected while levels of reinfection are unequal (i.e., *σ*_1_ < *σ*_2_ = *σ*_3_) results in Fig 8D which is qualitatively similar to when reactivation mechanisms are included (see Fig 8C). This observation suggests that reactivation pathways do not play a significant role when it comes to determining the outcome of treatment of late LTBI; rather it is the rate of reinfection and particularly reinfection of individuals treated with IPT that greatly influence treatment outcome.

## Discussion

In this study, a mathematical TB model accounting for heterogeneity in susceptibility to reinfection has been proposed. Unlike previous work, we distinguish four different levels of susceptibility to infection among the disease-free population. Analysis of the model yielded the following results:

1. Reinfection of previously infected individuals can lead to unusual dynamics, such as backward bifurcation and hysteresis effects. This is epidemiologically important because it could lead to extreme changes in disease burden following relatively minor epidemiological changes. The backward bifurcation effect is likely to be particularly important for TB, which is or has been endemic in virtually all regions of the world, but with decreasing burden. Therefore, abrupt declines in burden could well be observed as TB prevalence declines towards the bifurcation point between endemic disease and disease-free equilibrium;
2. It was found that the risk of reinfection among individuals treated with IPT (i.e., *σ*_2_) plays a central role in qualitative changes in model dynamics, particularly in shifting TB prevalence between low and high burden. In contrast, the risk of reinfection (i.e., *σ*_3_) among the recovered cohort who were previously treated from active TB (or self-cure) plays an insignificant role in terms of the qualitative dynamics of the model;
3. Treatment of early latent infection is universally beneficial irrespective of the level of reinfections among cohorts subject to reinfection. That is, treatment of early latent TB decreases TB prevalence. The benefit is strongest if individuals treated with IPT have a relatively low risk of reinfection i.e., compared to late LTBI. That is, when *σ*_2_ < *σ*_1_. (See eg., Fig 6B or 6D);
4. Similar to previous findings [36], the assumption that treatment decreases the risks of reinfection among both cohorts of individuals treated with IPT and individuals recovered from active TB was considered. Under this assumption, treatment of late latent infection has a consistently positive impact (see Fig 7A) and therefore may be highly synergistic with other interventions;
5. Alternatively, the assumption that treatment of late latent infection increases the risk of reinfection among IPT-treated individuals yields contrasting results to case (iii) above. That is, treatment of late LTBI increases TB prevalence in most settings, although treatment is more detrimental above the reinfection threshold, particularly in an intermediate prevalence zone lying between RT2 and RT1, as shown in Fig 7D;
6. Assume now that treatment of late latently infected individuals increases the risk of reinfection for individuals treated with IPT (i.e., *σ*_2_ > *σ*_1_) but decreases the risks of reinfection among recovered individuals (i.e., *σ*_3_ < *σ*_1_). Our results still show that increasing treatment of late LTBI increases the prevalence of active TB (see Fig 7C). This observation suggests that the level of immunity/susceptibility for patients who have previously received preventive therapy is the key quantity determining whether treatment will decrease or increase prevalence of active TB. Future research should focus on quantifying the value of this key epidemiological parameter (i.e., *σ*_2_);
7. It is observed that reactivation mechanisms (in particular reactivation from late latent infection (*η*) and from recovered individuals (*ω*)) play a minimal role in determining treatment outcomes, except in very low burden settings (i.e., a prevalence below 10^−4^ or 10 cases per 100,000 population).

Note that Gomes et al. [36] argued that the qualitative effects presented in their paper were generally robust against reasonable changes in parameter values and model assumptions. Gomes et al. assumed that individuals who were previously treated for active TB and individuals treated with preventive therapy are not differentiable and therefore can be treated as a single group. In particular both *σ*_2_ and *σ*_3_ were coupled as a single parameter, obscuring the understanding of the role of each type of immunity. However, our study shows that relaxing this assumption yields new epidemiological insights. This follows from the fact that it is impossible to tell from Gomes et al. whether an increase in prevalence of active TB is attributable to reinfection of previously treated individuals or those who have been treated with IPT. Thus, epidemiological studies able to quantify *σ*_2_ accurately would be important in shedding light on TB dynamics.

### WHO TB burden estimates

TB is present in every region and country of the world but its distribution varies greatly with the most highly endemic countries reporting rates of disease around 1000 per 100,000 per year, while the least endemic countries have rates as low as 5 per 100,000 per year. There can be little doubt that this observation of 200-fold differences in disease burden relates in part to heterogeneity in socio-economic development, living conditions, prevalence of comorbidities and the strength of health systems. However, given such a huge gulf in disease rates, additional factors may well be at work. Here we postulate that decreased susceptibility to reinfection in comparison to first infection acts to create a threshold effect, which can lead to a 100-fold increase in burden once crossed. Similarly, disease may be considerably easier to control once prevalence has dropped below the reinfection threshold and enters the low endemic, controllable zone. That is, while socio-economic development and improvements in treatment programs could explain gradual decreases in burden, this additional phenomenon may help to explain more dramatic shifts. For example, the recent rapid declines in TB burden in China and other countries of East Asia could be partly attributable to this threshold effect.

**Fig 9:**
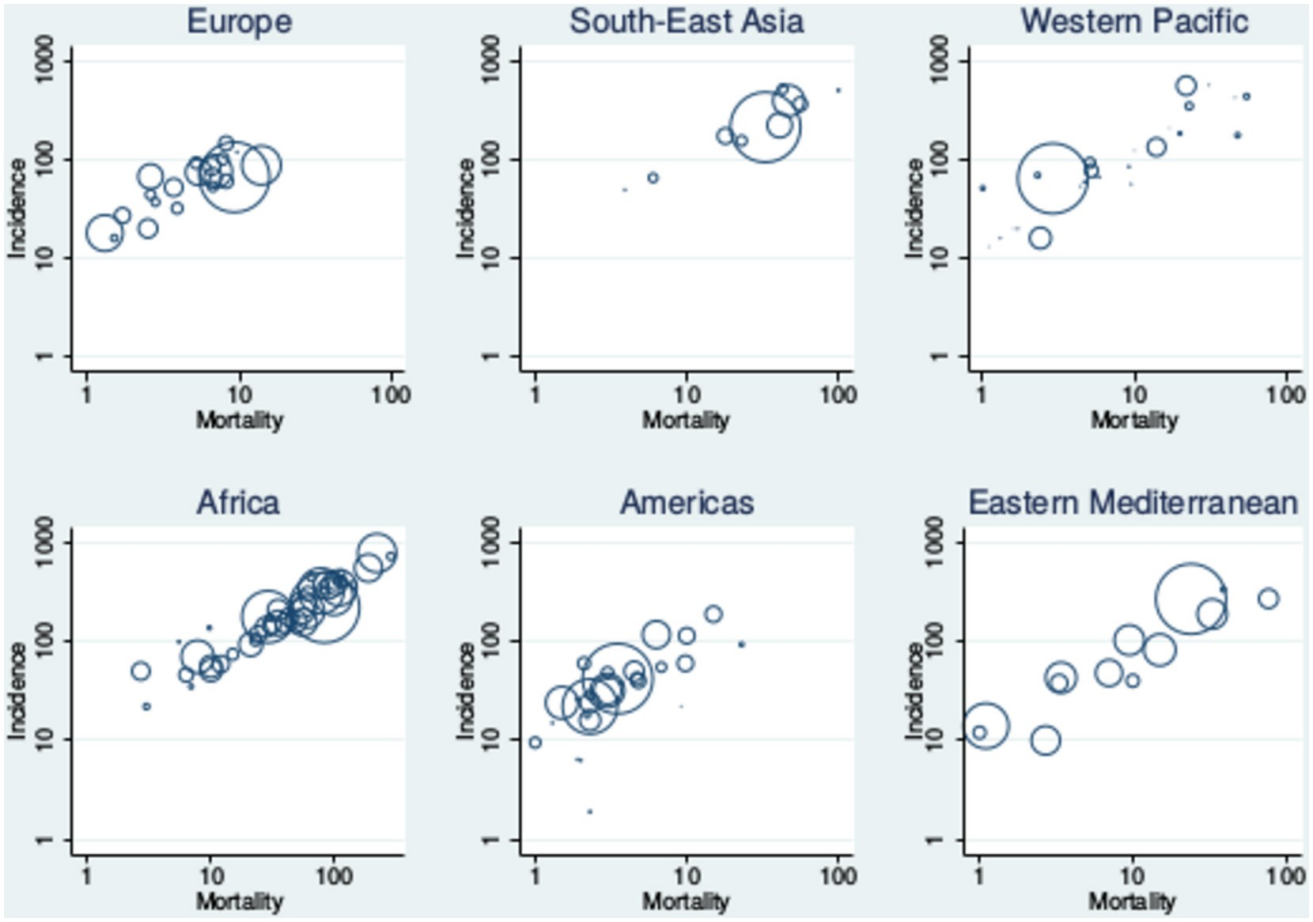
Illustration of world TB burden by WHO region obtained from the global TB Report data [6]. The size of the circle is proportional to the population of the country. Incidence is plotted relative to mortality not to illustrate the relationship between these two quantities, but rather as two complementary illustrations of disease burden. Clustering of countries into high and low burden groups is arguably observed to some degree, although this may be obscured by other epidemiological effects.

**Fig 10:**
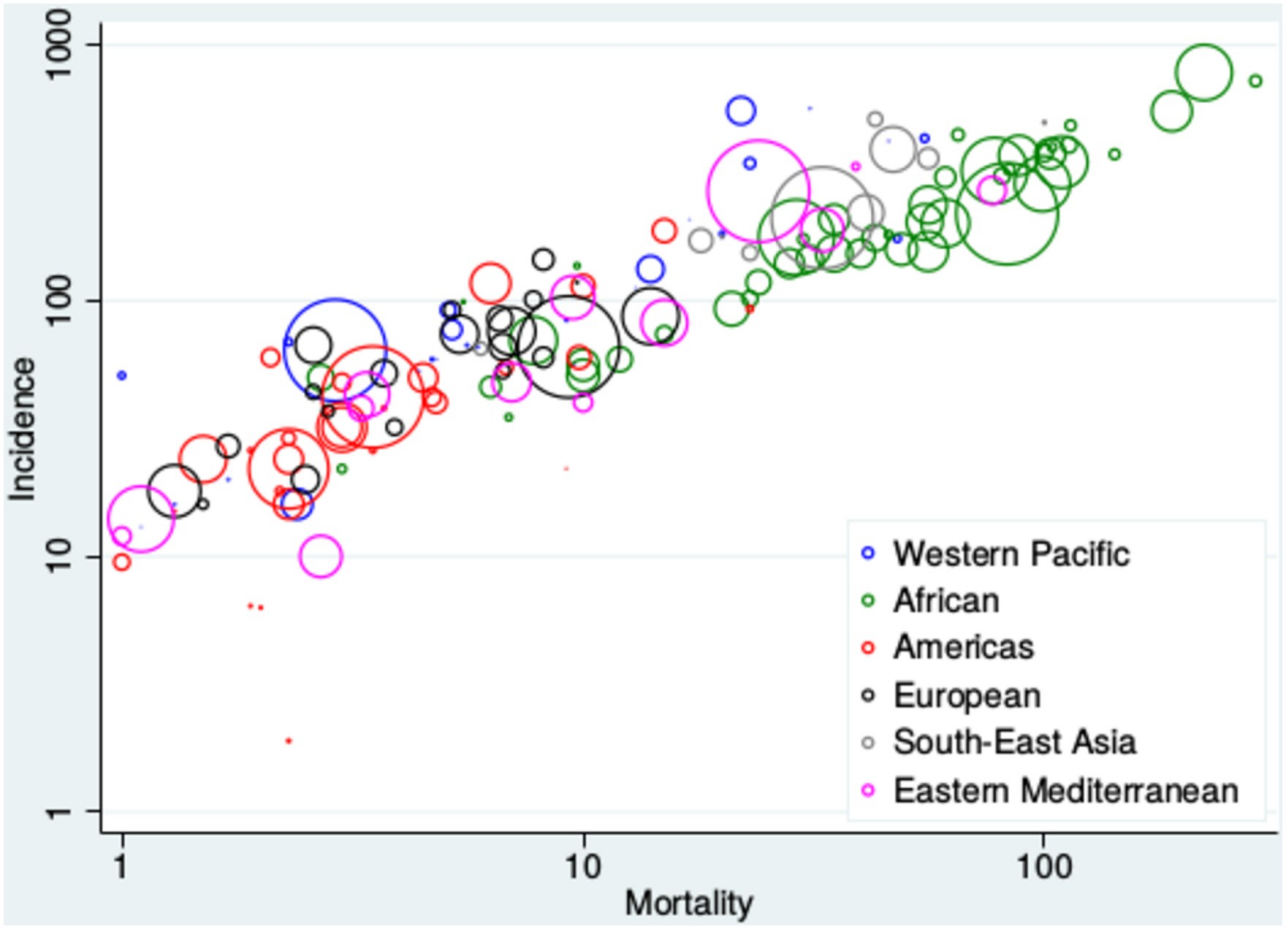
Illustration of global TB burden by country with colouring by region using global TB report data [6]. Note again that the diameter of the circle is proportional to the population of the country.

Empirical evidence for the role of reinfection heterogeneity is difficult to find, given high-quality data on TB burden has only recently become available. However, many regions of the world appear to show significant divides between high and low burden countries (Fig 9). Although this is not clearly apparent in all regions and an overall threshold is not evident (Fig 10), this grouping of countries is arguably seen in current WHO data [6]. The absence of a clear divide could relate to factors such as comorbidities (e.g., HIV infection), differences in health systems and socio-economic development, as well as the fact that TB transmission frequently occurs over a much smaller scale than a nation state. Therefore, the reported overall burden for individual countries actually represents the summation of many heterogeneous sub-epidemics, particularly for large countries such as China and India.

### Limitations and conclusion

Mathematical modelling is an important tool for epidemiologists since it provides insights where empirical epidemiological observations cannot. With the proposed model, the key parameter influencing preventive treatment outcomes is identified. It was found that the reinfection parameter accounting for reinfection of individuals treated with IPT (*σ*_2_), and not the parameter accounting for reinfection of recovered individuals (*σ*_3_), alters treatment outcome. Further, changes in *σ*_2_ can either be beneficial or detrimental when there are treatment programs. The need to quantify the parameter is important if epidemiologists are to accurately estimate its effect on TB dynamics. Moreover, it is observed that reactivation mechanisms (in particular reactivation from late latent infection and from recovered individuals) play a minimal role in determining treatment outcomes except in very low endemic settings.

Limitations of our model include that individuals being treated from early and late latent compartments are coalesced into a single compartment so as to simplify the model (although the effect of distinguishing these groups following preventive treatment has been previously explored [46]). This could be addressed in conjunction with our approach of distinguishing four susceptibility categories. Moreover, increased epidemiological realism could be incorporated, including more accurate parameterisation to specific settings. For example incomplete efficacy of preventive treatment could be reasonably considered and explored within the context of the model presented here as a proportional reduction in treatment rates.

Also, it is important to note that at this time it is unknown whether treatment with IPT confers additional immunity or removes the pre-existing immunity that we know people gain from untreated LTBI [35]. Some TB-family exposures increase immunity (e.g. BCG-vaccination, LTBI), whereas others appear to increase susceptibility (past episodes of disease) at the population level. From an immunological perspective, much is still not fully understood about the dynamic interaction between host and pathogen. Therefore, it is plausible that immunity could be increased or decreased. Whether this leads to more severe or milder disease is also uncertain. Although resistance to antituberculous agents is associated with poorer treatment outcomes [64], IPT-treated individuals do not tend to have higher rates of drug resistance at the population level; rather MDR-TB emerges in settings that have had poor historical programmatic performance, which are typically ones that have not used IPT widely. Nevertheless, our modelling study shows the necessity of trying to learn the impact of preventive therapy on immunity.

Finally, the proposed model is more applicable to high burden settings, as transmission in low-burden settings is limited. However, the use of IPT in high-burden settings is a critically important issue in global TB control. For instance see Ragonnet et al. [46] who strongly advocate for widespread use of IPT in high-burden settings. There is also increasing interest in this programmatic intervention, as WHO recommends expanding IPT in settings with an incidence of up to 100 per 100,000, which is not low in the global context (the global rate being 142 per 100,000 [43, 45, 46]).

## Supporting information

S1 Appendix Basic properties and computation of *R*_0_ for model Eq (1). (PDF)

S2 Appendix Interpretation for *R*_0_ of model Eq (1). (PDF)

S3 Appendix Non trivial steady states for model Eq (1). (PDF)

S4 Appendix Computation of reinfection thresholds. (PDF)
