# Supplementary material for: Modelling heterogeneity in host susceptibility to tuberculosis and its effect on public health interventions
Source: PLoS One. 2018 Nov 14;13(11):e0206603. doi: 10.1371/journal.pone.0206603 (PMC6235601; doi:10.1371/journal.pone.0206603)
Supplement: S1 Appendix — (PDF) [file pone.0206603.s001.pdf]

S1 Appendix. Basic properties and computation of R_0 for model equation (1).

The following system of nonlinear ordinary differential equations govern the model:

$$\begin{aligned}
\frac{dS}{dt} &= \lambda - \mu S - \beta IS, \\
\frac{dL_1}{dt} &= \sigma_1 \beta I L_2 + \sigma_2 \beta IP + \sigma_3 \beta IR + \beta IS - (\theta + \mu + \phi) L_1, \\
\frac{dL_2}{dt} &= (1-f)\phi L_1 - (\mu + \eta + \rho + \sigma_1 \beta I) L_2, \\
\frac{dI}{dt} &= \phi f L_1 + \eta L_2 + \omega R - (\mu + d + \tau + \alpha) I, \\
\frac{dP}{dt} &= \theta L_1 + \rho L_2 - (\mu + \sigma_2 \beta I) P, \\
\frac{dR}{dt} &= (\tau + \alpha) I - (\mu + \omega + \sigma_3 \beta I) R.
\end{aligned} \tag{1}$$

Note that all model parameters and variables of model equation (1) are considered to be non-negative since the model represents a human population. First a biologically and epidemiologically feasible region is established through the following theorem:

Theorem 0.1 *The region*

$$\Lambda := \{(S, L_1, L_2, I, P, R) \in \mathbb{R}^6_+ : \; S + L_1 + L_2 + I + P + R \leq 1\}$$

is positively-invariant and absorbing with respect to the model equation (1) *with initial conditions in* $\mathbb{R}^6_+$.

Proof: The proof involves showing that the feasible solution of model equation (1) are uniformly bounded in the region Λ. Supposing S, L_1, L_2, I, P, R is any solution of model equation (1) with positive initial conditions, then the total population fulfils the following inequality

$$\frac{dN}{dt} \leq \mu - \mu N. \tag{2}$$

It follows from (2) that $\frac{dN}{dt} \leq \mu - \mu N$ implies that $\frac{dN}{dt} \leq 0$ if $N \geq 1$. Applying the standard comparison theorem [1] it can be shown that

$$N(t) \leq N(0) e^{-\mu t} + (1 - e^{-\mu t}).$$

Specifically, $N(t) \leq 1$, if $N(0) \leq 1$ for all $t > 0$. Hence, under the flow induced by equation (1), the region Λ is positively invariant. Moreover, for $N(0) > 1$, the trajectory solutions $N(t)$ enters either in the region Λ finite time or asymptotically approaches 1. Thus, in the region Λ model equation (1) is considered to be mathematically and epidemiologically well posed [2] and the solution of all the trajectories generated by model (1) are in a biologically feasible region Λ.

The model system (1) has two important steady states: the disease free equilibrium and the non-trivial endemic equilibria where TB is expected to persist. By setting all the ever-infected states of model (1) to zero (i.e., $\mathrm{L}_1, \mathrm{L}_2, \mathrm{I}, \mathrm{P}, \mathrm{R} = 0$) the disease free equilibrium denoted by Y_0 is given as

$$Y_0 = (1, 0, 0, 0, 0, 0).$$

To compute the basic reproduction number the next generation operator (NGO) approach developed by Van den Driessche and Watmough [3] is applied. In order to use the method it is important to distinguish new infections in each class from all other changes in population. In model (1) the infected classes are $\mathrm{L}_1, \mathrm{L}_2, \mathrm{I}, \mathrm{P}, \mathrm{R}$. The system (1) can be written as

$$\dot{Y} = \mathcal{F}(Y) - \mathcal{V}(Y), \tag{3}$$

where $Y = (S,\ L_1,\ L_2,\ I,\ P,\ R)$. $\mathcal{F}(Y)$ represents the rate of appearance of new infections and $\mathcal{V}$ represents the rate of transition from one compartment to another. Thus,

$$\mathcal{F}(Y) = (0,\ \beta IS,\ 0,\ 0,\ 0,\ 0)^T.$$

The model system (1) has an intrinsic disease free equilibrium given as $Y_0 = (1,\ 0,\ 0,\ 0,\ 0,\ 0)$. Now the derivatives of $\mathcal{F}(Y)$ and $\mathcal{V}(Y)$ with respect to the infected compartment, evaluated at disease free equilibrium can respectively, be obtained as

$$F = \begin{bmatrix} 0 & 0 & \beta & 0 & 0 \\ 0 & 0 & 0 & 0 & 0 \\ 0 & 0 & 0 & 0 & 0 \\ 0 & 0 & 0 & 0 & 0 \\ 0 & 0 & 0 & 0 & 0 \end{bmatrix},\ V = \begin{bmatrix} k_1 & 0 & 0 & 0 & 0 \\ -(1-f)\phi & k_2 & 0 & 0 & 0 \\ -\phi f & -\eta & k_3 & 0 & -\omega \\ -\theta & -\rho & 0 & \mu & 0 \\ 0 & 0 & -(\tau+\alpha) & 0 & k_4 \end{bmatrix}$$

with

$$V^{-1} = \begin{bmatrix} \frac{1}{k_1} & 0 & 0 & 0 & 0 \\ k_{21} & \frac{1}{k_2} & 0 & 0 & 0 \\ k_{31} & k_{32} & k_{33} & 0 & k_{35} \\ k_{41} & \frac{\rho}{\mu k_2} & 0 & \frac{1}{\mu} & 0 \\ k_{51} & k_{52} & k_{53} & 0 & k_{55} \end{bmatrix},$$

where

$$\begin{aligned}
k_1 &= (\theta + \mu + \phi), \\
k_2 &= (\mu + \eta + \rho), \\
k_3 &= (\mu + d + \tau + \alpha), \\
k_4 &= (\mu + \omega), \\
k_{21} &= \frac{(1-f)\phi}{k_1 k_2}, \\
k_{31} &= \frac{(f\phi(\mu+\rho) + \eta\phi)k_4}{k_1 k_2((\mu+d)k_4 + \mu(\tau+\alpha))}, \\
k_{32} &= \frac{\eta k_4}{k_2((\mu+d)k_4 + \mu(\tau+\alpha))}, \\
k_{33} &= \frac{k_4}{(\mu+d)k_4 + \mu(\tau+\alpha)}, \\
k_{35} &= \frac{\omega}{(\mu+d)k_4 + \mu(\tau+\alpha)}, \\
k_{41} &= \frac{k_3(\mu\theta k_1 + \mu\rho\phi(1-f)) + (\mu+d)\omega(\theta k_2 + \rho\phi(1-f))}{\mu k_1 k_2((\mu+d)k_4 + \mu(\tau+\alpha))}, \\
k_{51} &= \frac{\phi(\tau+\alpha)(f(\mu+\rho)+\eta)}{k_1 k_2((\mu+d)k_4 + \mu(\tau+\alpha))}, \\
k_{52} &= \frac{\eta(\tau+\alpha)}{k_2((\mu+d)k_4 + \mu(\tau+\alpha))}, \\
k_{53} &= \frac{(\tau+\alpha)}{((\mu+d)k_4 + \mu(\tau+\alpha))}, \\
k_{55} &= \frac{k_3}{((\mu+d)k_4 + \mu(\tau+\alpha))}.
\end{aligned}$$

Following Van den Driessche and Watmough [3] the basic reproduction number is defined as the spectral radius of the next generation matrix, FV^{-1} (i.e., $R_0 = \bar{\rho}(FV^{-1})$, where $\bar{\rho}$ denote the spectral radius), which for the model system (1) is given as

$$R_0 = \frac{\beta(\mu+\omega)(f\phi(\mu+\rho)+\eta\phi)}{((\mu+d)(\mu+\omega)+\mu(\tau+\alpha))(\mu+\eta+\rho)(\theta+\mu+\phi)}. \quad (4)$$

References

[1] Smith HL. The theory of the chemostat: dynamics of microbial competition. Cambridge university press. 1995; volume 13.

[2] Hethcote, HW. The mathematics of infectious diseases. SIAM Review. 2000; 42(4):599–653.

[3] Van den Driessche P, Watmough J. Reproduction numbers and subthreshold endemic equilibria for compartmental models of disease transmission. Mathematical biosciences. 2002;180(1):29–48.
