# Supplementary material for: Modelling heterogeneity in host susceptibility to tuberculosis and its effect on public health interventions
Source: PLoS One. 2018 Nov 14;13(11):e0206603. doi: 10.1371/journal.pone.0206603 (PMC6235601; doi:10.1371/journal.pone.0206603)
Supplement: S2 Appendix — (PDF) [file pone.0206603.s002.pdf]

S2 Appendix. Interpretation for R_0 of model equation (1).

Consider,

$$R_0 = \frac{\beta(\mu+\omega)\phi(f(\mu+\rho)+\eta)}{((\mu+d)(\mu+\omega)+\mu(\tau+\alpha))(\mu+\eta+\rho)(\theta+\mu+\phi)}. \quad (1)$$

The expression for basic reproduction number R_0 as given above in equation (1) can be decoupled to be expressed as a combination of various pathways leading to active TB. First starting with a simpler scenario where treated active TB individuals do not reactivate. That is by setting $\omega = 0$, the R_0 expression (1) can be rewritten as

$$R_0' = \left(\frac{\beta}{\mu+d+\tau+\alpha}\right)\left[\left(\frac{f\phi}{\mu+\theta+\phi}\right)+\left(\frac{(1-f)\phi}{\mu+\theta+\phi}\right)\left(\frac{\eta}{\mu+\eta+\rho}\right)\right]. \quad (2)$$

Each factor in R_0' has an epidemiological interpretation as follows:

(i) $\frac{\beta}{\mu+d+\tau+\alpha}$ represent the average number of secondary cases an individual with active TB produces;

(ii) $\left(\frac{f\phi}{\mu+\theta+\phi}\right)$ reflect that individuals progress toward active TB through the early latent compartment;

(iii) $\left(\frac{(1-f)\phi}{\mu+\theta+\phi}\right)\left(\frac{\eta}{\mu+\eta+\rho}\right)$, reflect that individuals progress to active TB through late latent compartment. Note that $\left(\frac{(1-f)\phi}{\mu+\theta+\phi}\right)$ account for the fraction that proceed to late latent class while $\left(\frac{\eta}{\mu+\eta+\rho}\right)$ is the probability of surviving the late latent compartment and progressing to active TB.

Now, considering that $\omega > 0$, recovered individuals have an additional chance to progress to active TB that is independent of re-exposures. This additional

contribution for the infectious period is given by the term

$$\Upsilon = 1 + \frac{(\tau+\alpha)}{\mu+d+\tau+\alpha}\frac{\omega}{\mu+\omega} + \left(\frac{(\tau+\alpha)}{\mu+d+\tau+\alpha}\frac{\omega}{\mu+\omega}\right)^2 + \cdots$$

$$= \frac{1}{1-\frac{(\tau+\alpha)}{\mu+d+\tau+\alpha}\frac{\omega}{\mu+\omega}}$$

$$= \frac{(\mu+d+\tau+\alpha)(\mu+\omega)}{\mu(\mu+d+\tau+\alpha)+\omega(\mu+d)} \quad (3)$$

that results from the innumerable chances the infected individual has to repeat this event. The full expression for R_0 is then obtained by the product of equations (2) and (3). That is

$$\begin{aligned} R_0 =& R_0'\Upsilon \\ =& \left(\frac{\beta}{\mu+d+\tau+\alpha}\right)\left[\left(\frac{f\phi}{\mu+\theta+\phi}\right)+\left(\frac{(1-f)\phi}{\mu+\theta+\phi}\right)\left(\frac{\eta}{\mu+\eta+\rho}\right)\right] \\ &\times\left(\frac{(\mu+d+\tau+\alpha)(\mu+\omega)}{\mu(\mu+d+\tau+\alpha)+\omega(\mu+d)}\right). \end{aligned}$$

References

[1] Smith HL. The theory of the chemostat: dynamics of microbial competition. Cambridge university press. 1995; volume 13.

[2] Hethcote, HW. The mathematics of infectious diseases. SIAM Review. 2000; 42(4):599–653.

[3] Van den Driessche P, Watmough J. Reproduction numbers and subthreshold endemic equilibria for compartmental models of disease transmission. Mathematical biosciences. 2002;180(1):29–48.
