# Supplementary material for: Modelling heterogeneity in host susceptibility to tuberculosis and its effect on public health interventions
Source: PLoS One. 2018 Nov 14;13(11):e0206603. doi: 10.1371/journal.pone.0206603 (PMC6235601; doi:10.1371/journal.pone.0206603)
Supplement: S3 Appendix — (PDF) [file pone.0206603.s003.pdf]

S3 Appendix. Non trivial steady states for model equation (1).

Due to the complex nature of the model system (1) the steady states cannot be explicitly expressed in terms of model parameters. Consequently, all steady states are written in terms of I^*. Namely

$$S^* = \frac{\mu + dI^*}{\mu + \beta I^*},$$

$$L_1^* = \frac{(\mu + \eta + \rho + \sigma_1 \beta I^*)((\mu + d + \tau + \alpha)(\mu + \sigma_3 \beta I^*) + \omega * (\mu + d))I^*}{(\mu + \omega + \sigma_3 \beta I^*)(f\phi(\mu + \rho + \sigma_1 \beta I^*) + \eta\phi)},$$

$$L_2^* = \frac{(1 - f)\phi((\mu + d + \tau + \alpha)(\mu + \sigma_3 \beta I^*) + \omega(\mu + d))I^*}{(\mu + \omega + \sigma_3 \beta I^*)(f\phi(\mu + \rho + \sigma_1 \beta I^*) + \eta\phi)},$$

$$P^* = \frac{((\mu + d + \tau + \alpha)(\mu + \sigma_3 \beta I^*) + \omega(\mu + d))(\theta(\mu + \eta + \rho + \sigma_1 \beta I^*) + \rho(1 - f)\phi)I^*}{(\mu + \sigma_2 \beta I^*)(\mu + \omega + \sigma_3 \beta I^*)(f\phi(\mu + \rho + \sigma_1 \beta I^*) + \eta\phi)},$$

$$R^* = \frac{(\tau + \alpha)I^*}{\mu + \omega + \sigma_3 \beta I^*}.$$

I^* can be solved from the following polynomial;

$$g(I^*) = I^*(d_4 I^{*4} + d_3 I^{*3} + d_2 I^{*2} + d_1 I^* + d_0) = 0,$$

where

$$d_4 = -\sigma_1\sigma_2\sigma_3\mu(f\phi + \mu + d + \tau + \alpha)\beta^4,$$

$$\begin{aligned}
d_3 = {} & \sigma_1\sigma_2\beta^3\mu(1-f)\ \phi(\mu + d + \tau + \alpha) + \sigma_1\sigma_2\sigma_3\mu(1-f)\phi\beta^3(\mu + d + \tau + \alpha) \\
& + \sigma_1\sigma_3\mu\beta^3(1-f)\phi(\mu + d + \tau + \alpha) + \sigma_1\sigma_2\beta^3(1-f)\phi\omega(\mu + d) \\
& + \sigma_1\sigma_2\sigma_3\mu\beta^3\theta(\mu + d + \tau + \alpha) \\
& + \sigma_2\sigma_3\beta^3\theta(\mu + \eta + \rho)(\mu + d + \tau + \alpha) + \sigma_1\sigma_2\theta\beta^3\mu(\mu + d + \tau + \alpha) \\
& + \sigma_1\sigma_2\theta\beta^3\omega(\mu + d) + \sigma_2\sigma_3\rho(1-f)\phi\beta^3(\mu + d + \tau + \alpha) + \sigma_1\sigma_2\sigma_3\mu\beta^3(\tau + \alpha)f\phi \\
& + \sigma_1\sigma_3\mu\beta^3(\tau + \alpha)f\phi + \sigma_2\sigma_3\beta^3(\tau + \alpha)(f\phi(\mu + \rho) + \eta\phi) + \sigma_1\sigma_3\mu d\beta^3 f\phi \\
& + \sigma_1\sigma_2 d(\mu + \omega)\beta^3 f\phi + \sigma_2\sigma_3 d\beta^3(f\phi(\mu + \rho) + \eta\phi) \\
& + \sigma_1\sigma_2\sigma_3\mu\beta^4 f\phi - \sigma_1\sigma_2\sigma_3\mu(\theta + \mu + \phi)\beta^3(\mu + d + \tau + \alpha) \\
& - \sigma_1\sigma_3\mu\beta^3(\mu + d + \tau + \alpha)(\theta + \mu + \phi) \\
& - \sigma_2\sigma_3\beta^3(\mu + \eta + \rho)(\mu + d + \tau + \alpha)(\theta + \mu + \phi) \\
& - \sigma_1\sigma_2\beta^3\mu(\theta + \mu + \phi)(\mu + d + \tau + \alpha) \\
& - \sigma_1\sigma_2\beta^3\omega(\mu + d)(\theta + \mu + \phi),
\end{aligned}$$

$$\begin{aligned}
d_2 = {} & \sigma_1\sigma_2\mu^2\beta^2(1-f)\phi(\mu + d + \tau + \alpha) + \sigma_1\beta^2\mu^2(1-f)\phi(\mu + d + \tau + \alpha) \\
& + \sigma_1\sigma_3\beta^2(1-f)\phi\mu^2(\mu + d + \tau + \alpha) + \sigma_1\sigma_2\beta^2(1-f)\phi\omega\mu(\mu + d) \\
& + \sigma_1\beta^2(1-f)\phi\omega\mu(\mu + d) + \sigma_2\sigma_3\mu\theta\beta^2(\mu + \eta + \rho)(\mu + d + \tau + \alpha) \\
& + \sigma_1\sigma_2\beta^2\mu^2\theta(\mu + d + \tau + \alpha) + \sigma_1\sigma_2\mu\theta\beta^2\omega(\mu + d) \\
& + \sigma_2\sigma_3\beta^2\mu\rho(1-f)\phi(\mu + d + \tau + \alpha) + \sigma_2\beta^2\theta(\mu + \eta + \rho)\mu(\mu + d + \tau + \alpha) \\
& + \sigma_2\beta^2\theta(\mu + \eta + \rho)\omega(\mu + d) + \sigma_2\beta^2\rho(1-f)\phi\mu(\mu + d + \tau + \alpha) \\
& + \sigma_2\beta^2\rho(1-f)\phi\omega(\mu + d) + \sigma_1\sigma_3\beta^2\mu^2(\tau + \alpha)f\phi \\
& + \sigma_3\beta^2(\tau + \alpha)\mu(f\phi(\mu + \rho) + \eta\phi) + \sigma_1\sigma_3\mu^2\beta^3 f\phi \\
& + \sigma_1\sigma_2\mu(\mu + \omega)\beta^3 f\phi + \sigma_2\sigma_3\mu\beta^3(f\phi(\mu + \rho) + \eta\phi) + \sigma_1\beta^2 d\mu(\mu + \omega)f\phi \\
& + \sigma_3\beta^2\mu d(f\phi(\mu + \rho) + \eta\phi) + \sigma_2 d\beta^2(\mu + \omega)(f\phi(\mu + \rho) + \eta\phi) \\
& + \sigma_2\sigma_3\mu\beta^2(\tau + \alpha)(f\phi(\mu + \rho) + \eta\phi) - \sigma_1\sigma_3\mu^2\beta^2(\mu + d + \tau + \alpha)(\mu + \phi + \theta) \\
& - \sigma_2\sigma_3\beta^2\mu(\theta + \mu + \phi)(\mu + \eta + \rho)(\mu + d + \tau + \alpha) \\
& - \sigma_1\sigma_2\beta^2\mu^2(\theta + \mu + \phi)(\mu + d + \tau + \alpha) - \sigma_1\sigma_2\beta^2\mu(\theta + \mu + \phi)\omega(\mu + d) \\
& - \sigma_3\beta^2\mu(\mu + \theta + \phi)(\mu + \eta + \rho)(\mu + d + \tau + \alpha) \\
& - \sigma_1\mu\beta^2(\theta + \mu + \phi)\omega(\mu + d) - \sigma_2\mu\beta^2(\theta + \mu + \phi)(\mu + \eta + \rho)(\mu + d + \tau + \alpha) \\
& - \sigma_1\beta^2\mu^2(\theta + \mu + \phi)(\mu + d + \tau + \alpha) - \sigma_2\beta^2(\theta + \mu + \phi)(\mu + \eta + \rho)\omega(\mu + d),
\end{aligned}$$

$$
\begin{aligned}
d_1 = {} & \sigma_1\beta(1-f)\phi\mu^3(\mu+d+\tau+\alpha) + \sigma_1\beta(1-f)\phi(\mu+d)\omega\mu^2 \\
& + \sigma_2\beta\mu^2\theta(\mu+\eta+\rho)(\mu+d+\tau+\alpha) + \sigma_2\beta\mu\theta(\mu+\eta+\rho)\omega(\mu+d) \\
& + \sigma_2\beta\mu^2\rho(1-f)\phi(\mu+d+\tau+\alpha) + \sigma_2\beta\mu\rho(1-f)\phi\omega(\mu+d) \\
& + \sigma_3\beta(\tau+\alpha)\mu^2(f\phi(\mu+\rho)+\eta\phi) + \sigma_1\beta^2\mu^2(\mu+\omega)f\phi \\
& + \sigma_3\beta^2\mu^2(f\phi(\mu+\rho)+\eta\phi) + \sigma_2\beta^2\mu(\mu+\omega)(f\phi(\mu+\rho)+\eta\phi) \\
& + \beta d\mu(\mu+\omega)(f\phi(\mu+\rho)+\eta\phi) - \sigma_3\beta\mu^2(\theta+\mu+\phi)(\mu+\eta+\rho)(\mu+d+\tau+\alpha) \\
& - \sigma_1\beta\mu^3(\theta+\mu+\phi)(\mu+d+\tau+\alpha) - \sigma_1\beta\mu^2(\theta+\mu+\phi)\omega(\mu+d) \\
& - \sigma_2\beta\mu^2(\theta+\mu+\phi)(\mu+\eta+\rho)(\mu+d+\tau+\alpha) \\
& - \sigma_2\beta\mu(\theta+\mu+\phi)(\mu+\eta+\rho)\omega(\mu+d) \\
& - \beta\mu^2(\theta+\mu+\phi)(\mu+\eta+\rho)(\mu+d+\tau+\alpha) \\
& - \beta\mu\omega(\theta+\mu+\phi)(\mu+d)(\mu+\eta+\rho),
\end{aligned}
$$

$$
\begin{aligned}
d_0 = {} & \beta\mu^2(\mu+\omega)(f\phi(\mu+\rho)+\eta\phi) \\
& - \mu^2(\theta+\mu+\phi)(\mu+\eta+\rho)((\mu+\omega)(\mu+d)+\mu(\tau+\alpha)).
\end{aligned}
$$
