# Supplementary material for: Modelling heterogeneity in host susceptibility to tuberculosis and its effect on public health interventions
Source: PLoS One. 2018 Nov 14;13(11):e0206603. doi: 10.1371/journal.pone.0206603 (PMC6235601; doi:10.1371/journal.pone.0206603)
Supplement: S4 Appendix — (PDF) [file pone.0206603.s004.pdf]

S4 Appendix. Computation of reinfection thresholds

Following Case (i), Case (ii) and Case (iii) as shown in the main text, the respective reinfection submodels of model equation (1) can be obtained as

$$\begin{aligned}
\frac{dL_2}{dt} &= \lambda + (1-f)\phi L_1 - (\mu + \rho + \sigma_1 \beta I)L_2, \\
\frac{dL_1}{dt} &= \sigma_1 \beta I L_2 - (\theta + \mu + \phi)L_1, \\
\frac{dI}{dt} &= \phi f L_1 - (\mu + d + \tau + \alpha)I.
\end{aligned} \tag{1}$$

$$\begin{aligned}
\frac{dP}{dt} &= \lambda + \theta L_1 - (\mu + \sigma_2 \beta I)P, \\
\frac{dL_1}{dt} &= \sigma_2 \beta I P - (\theta + \mu + \phi)L_1, \\
\frac{dI}{dt} &= \phi f L_1 - (\mu + d + \tau + \alpha)I.
\end{aligned} \tag{2}$$

$$\begin{aligned}
\frac{dR}{dt} &= \lambda + (\tau + \alpha)I - (\mu + \sigma_3 \beta I)R, \\
\frac{dL_1}{dt} &= \sigma_3 \beta I R - (\theta + \mu + \phi)L_1, \\
\frac{dI}{dt} &= \phi f L_1 - (\mu + d + \tau + \alpha)I.
\end{aligned} \tag{3}$$

The reinfection threshold for the first reinfection submodel (1) can be computed following the procedure followed for the reinfection submodel for a scenario where $\sigma_1 = \sigma_2 = \sigma_3$, (see the text) and it can be found that the reinfection submodel sustains an endemic equilibrium when transmission is above the critical value given in equation (5). Consequently, the reinfection threshold written in terms of R_0 is similar to equation (6). The other, reinfection thresholds, RT2 and RT3 are obtained from their respective reinfection submodels (9) and (10) as

$$\begin{aligned}
RT2 &= \frac{1}{\sigma_2}\frac{(\theta + \mu + \phi)(\mu + d + \tau + \alpha)}{\phi f}, \\
RT3 &= \frac{1}{\sigma_3}\frac{(\theta + \mu + \phi)(\mu + d + \tau + \alpha)}{\phi f}.
\end{aligned}$$

Similar to RT1, both RT2 and RT3 represent a transmission rate above which disease prevalences steeply increases. The reinfection thresholds RT2

and RT3 expressed in terms of R_0 are respectively, given as

$$R_0^{RT2} = \frac{1}{\sigma_2}\frac{(\mu+d+\tau+\alpha)(\mu+\omega)(f\phi(\mu+\rho)+\eta\phi)}{f\phi((\mu+d)(\mu+\omega)+\mu(\tau+\alpha))(\mu+\eta+\rho)}, \tag{4}$$

$$R_0^{RT3} = \frac{1}{\sigma_3}\frac{(\mu+d+\tau+\alpha)(\mu+\omega)(f\phi(\mu+\rho)+\eta\phi)}{f\phi((\mu+d)(\mu+\omega)+\mu(\tau+\alpha))(\mu+\eta+\rho)}. \tag{5}$$

Endemic equilibria of the reinfection submodel (3) (see text)

Let $H^* = L_2^* + P^* + R^*$ then, the equilibrium of the reinfection submodel (3) (in the text) can be easily obtained by setting the right-hand terms to zero and evaluating for H^*, L_1^* and I^* as

$$H^* = \frac{(\theta+\mu+\phi)(\mu+d+\tau+\alpha)}{f\phi\sigma_1\beta}, \tag{6}$$

$$L_1^* = \frac{(\mu+d+\tau+\alpha)I^*}{f\phi}, \tag{7}$$

$$I^* = \frac{f\phi\left[\beta - \frac{(\theta+\mu+\phi)(\mu+d+\tau+\alpha)}{\sigma_1 f\phi}\right]}{\beta[f\phi+(\mu+d+\tau+\alpha)]}. \tag{8}$$
